# Aluminum-copper alloy anode materials for high-energy aqueous aluminum batteries

**DOI:** 10.1038/s41467-022-28238-3

**Published:** 2022-01-31

**Authors:** Qing Ran, Hang Shi, Huan Meng, Shu-Pei Zeng, Wu-Bin Wan, Wei Zhang, Zi Wen, Xing-You Lang, Qing Jiang

**Affiliations:** 1 Key Laboratory of Automobile Materials (Jilin University), Ministry of Education, School of Materials Science and Engineering, and Electron Microscopy Center, Jilin University, Changchun, 130022 China; 2 State Key Laboratory of Automotive Simulation and Control, Jilin University, Changchun, 130022 China

**Keywords:** Batteries, Materials for energy and catalysis, Electrochemistry, Energy storage

## Abstract

Aqueous aluminum batteries are promising post-lithium battery technologies for large-scale energy storage applications because of the raw materials abundance, low costs, safety and high theoretical capacity. However, their development is hindered by the unsatisfactory electrochemical behaviour of the Al metal electrode due to the presence of an oxide layer and hydrogen side reaction. To circumvent these issues, we report aluminum-copper alloy lamellar heterostructures as anode active materials. These alloys improve the Al-ion electrochemical reversibility (e.g., achieving dendrite-free Al deposition during stripping/plating cycles) by using periodic galvanic couplings of alternating anodic α-aluminum and cathodic intermetallic Al_2_Cu nanometric lamellas. In symmetric cell configuration with a low oxygen concentration (i.e., 0.13 mg L^−1^) aqueous electrolyte solution, the lamella-nanostructured eutectic Al_82_Cu_18_ alloy electrode allows Al stripping/plating for 2000 h with an overpotential lower than ±53 mV. When the Al_82_Cu_18_ anode is tested in combination with an Al_*x*_MnO_2_ cathode material, the aqueous full cell delivers specific energy of ~670 Wh kg^−1^ at 100 mA g^−1^ and an initial discharge capacity of ~400 mAh g^−1^ at 500 mA g^−1^ with a capacity retention of 83% after 400 cycles.

## Abstract (web summary)

Aqueous Al-ion batteries are attractive post-lithium battery technologies. Here Al-Cu alloy lamellar heterostructures with periodic galvanic couplings are reported as efficient anode active material to produce high-energy aqueous Al-ion batteries.

## Introduction

Safe and reliable large-scale energy storage technologies are indispensable for many emerging applications including electric vehicles and grid integration of intermittent renewable energy sources^1,2^. Although lithium-ion batteries (LIBs) dominate the present energy-storage landscape, they are far from meeting the needs of large-scale energy storage due to their inherent issues such as high cost and scarcity of lithium resources, as well as safety problems associated with highly toxic and flammable organic electrolytes^2–4^. This dilemma has led to the recent boom in the development of alternative battery technologies^2,5^, especially aqueous rechargeable batteries that use monovalent (Na^+^^6^, K^+^^7^) or multivalent (Mg^2+^^8,9^, Al^3+^^10–13^, Ca^2+^^15^, Zn^2+^^16–20^) cations as charge carriers in low-cost and safe water-based electrolytes^21,22^. Among these post-lithium energy storage devices, aqueous rechargeable aluminum-metal batteries (AR-AMBs) hold great promise as safe power sources for transportation and viable solutions for grid-level energy storage because of metallic aluminum (Al) offering high volumetric/gravimetric capacities (8056 mAh cm^−3^ and 2981 mAh g^−1^) by a three-electron redox reaction^10,13,21,23–26^, in addition to its low cost and high Earth abundance^10,21^. Despite various cathode materials including titanium oxides^27,28^, bismuth oxides^29^, vanadium oxides^30^, aluminum manganese oxides^12,15,22,31^, and Prussian blue analogues^32,33^ have been explored for reversible Al^3+^ storage/delivery in aqueous electrolytes via intercalation or conversion reaction mechanisms^10,13,22^, these AR-AMBs generally exhibit low Coulombic efficiency and inadequate cycling stability, even in water-in-salt aluminum trifluoromethanesulfonate (Al(OTF)_3_) electrolytes^10–12,22–25^. Their poor rechargeability primarily results from irreversibility of Al anode due to inherent formation of the insulating and passivating aluminum oxide (alumina) layer that substantially limits Al^3+^ transportation for subsequent Al stripping/plating^10,11,22–25,34^. While increasing potentials to drive ion transport through such alumina layer, there concomitantly take place hydrogen evolution reaction and corrosion reaction to continuously deplete aqueous electrolyte and Al anode^10,11,23,24^. Despite the native oxide layer could be moderated by alloying of Al and small amount of other elements^14,23,24^ or by constructing artificial solid electrolyte interphases^11,35^, these ineluctable side reactions essentially impede widespread implementation of aqueous aluminum-metal batteries as a rechargeable energy-storage technology for practical use. Therefore, it is highly desirable to explore feasible strategies to improve Al reversibility of Al-based anode materials for high-performance AR-AMBs.

Here we demonstrate that eutectic engineering of Al-based alloy anodes improves their Al reversibility in aqueous electrolyte, based on eutectic Al_82_Cu_18_ (at%) alloy (E-Al_82_Cu_18_) with a lamellar nanostructure consisting of alternating α-Al and intermetallic Al_2_Cu nanolamellas. Such nanostructure enlists the E-Al_82_Cu_18_ electrode to have periodically localized galvanic couples of anodic α-Al and cathodic Al_2_Cu by making use of their distinct corrosion potentials (−1.65 V and −1.2 V versus H^+^/H_2_)^36,37^. Therein, the more-noble Al_2_Cu lamellas serve as electron transfer pathway to facilitate Al stripping from the constituent less-noble Al lamellas and work as nanopatterns to guide subsequent dendrite-free Al plating, enabling improved Al reversibility at low potentials especially in an aqueous Al(OTF)_3_ electrolyte with a low oxygen concentration of 0.13 mg L^−1^, which significantly inhibits hydrogen evolution reaction and further formation of the passivating oxide layer. As a result, the E-Al_82_Cu_18_ electrodes exhibit improved Al stripping/plating behaviors, with the overpotential of as low as ~53 mV and the Coulombic efficiency of ~100%, for more than 2000 h. When assembled with Al_*x*_MnO_2_ cathode, the E-Al_82_Cu_18_ electrodes render full cells to achieve high specific energy of ~670 Wh kg^−1^ or energy density of 815 Wh L^−1^ at 100 mA g^−1^ (based on the loading mass of Al_*x*_MnO_2_ or the volume of cathode), and retain 83% capacity after 400 cycles. The facile and scalable metallurgical technology of eutectic engineering opens a way to develop high-performance alloy anodes for next-generation aqueous rechargeable metal batteries.

## Results

### Physicochemical characterizations of the Al-Cu alloys

**Fig. 1:**
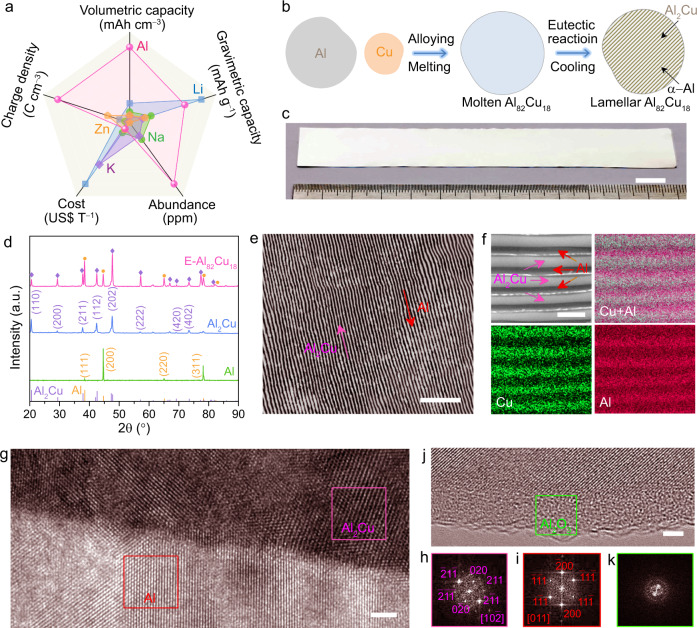
Merits of Al metal anode and microstructure characterizations of eutectic Al-Cu alloys. **a** Comparisons of electrochemical properties, cost, and abundance for Al, Zn, K, Na, and Li. **b** Schematic illustrating the procedure to prepare lamella-nanostructured E-Al_82_Cu_18_ alloy composed of alternating α-Al (gray) and intermetallic Al_2_Cu (dark yellow) lamellas. **c** Photograph of as-prepared E-Al_82_Cu_18_ alloy sheets with dimensions of ~13 cm × ~1.5 cm × ~400 μm. Scale bar, 1 cm. **d** XRD patterns of E-Al_82_Cu_18_ (pink line), intermetallic Al_2_Cu (blue line) and monometallic Al (green line) electrode foils. The line patterns show reference cards 04–0787 and 25–0012 for face-centered cubic Al (yellow lines) and body-centered tetragonal Al_2_Cu (blue lines) according to JCPDS, respectively. **e** Representative optical micrograph of lamella-nanostructured E-Al_82_Cu_18_ alloy with an interlamellar spacing of ~420 nm. Scale bar, 5 μm. **f** SEM backscattered electron image of E-Al_82_Cu_18_ with different contrasts corresponding to α-Al and intermetallic Al_2_Cu lamellas, as well as the corresponding EDS elemental mapping of Cu (in green) and Al (in red). Scale bar, 1 μm. **g** HRTEM image of E-Al_82_Cu_18_ at Al_2_Cu/Al interfacial region. Scale bar, 2 nm. **h**, **i** FFT patterns of selected red and pink boxes in intermetallic Al_2_Cu (**h**) and metallic Al (**i**) phases. **j** HRTEM image of Al/Al_2_O_3_ interfacial region. Scale bar, 2 nm. **k** FFT patterns of the selected area in amorphous Al_2_O_3_ layer in **j**.

Al metal is one of the most attractive anode materials in post-lithium batteries in view of its numerous merits, such as low cost and high Earth abundance, as well as high charge density and gravimetric/volumetric capacities, compared with Na, K, and Zn **(**Fig. 1a and Supplementary Table 1)^10,21,24,25^. To tackle its inherent irreversibility issue due to the oxide layer, here we design periodically aligned metallic/intermetallic Al/Al_2_Cu galvanic couples in E-Al_82_Cu_18_ alloy to improve the Al stripping/plating in AR-AMBs, distinguishing from eutectic Zn-Sn alloy to minimize active materials pulverization and subsequent loss of electrical contact in LIBs^38^, and eutectic Zn-Al alloy to address dendrite issue of Zn metal anode in aqueous rechargeable zinc-ion batteries^39^. With the assumption that all Al atoms can take part in the electrochemical stripping/plating, the theoretical volumetric and gravimetric capacities of the E-Al_82_Cu_18_ alloy are estimated to reach 7498 mAh cm^−3^ and 1965 mAh  g^−1^.

The E-Al_82_Cu_18_ alloy is prepared by arc-melting pure Al (99.994%) and Cu (99.996%) metals with a eutectic composition of 82:18 (at%), followed by a water cycle-assisted furnace cooling for the formation of immiscible α-Al and Al_2_Cu eutectoid via an eutectic solidification reaction (Fig. 1b, c)^40,41^. X-ray diffraction (XRD) characterization demonstrates the spontaneously separated α-Al and Al_2_Cu phases in the as-prepared E-Al_82_Cu_18_ alloy (Fig. 1d), with two sets of characteristic XRD patterns corresponding to the (111), (200), (220), and (311) planes of face-centered cubic (fcc) Al metal (JCPDS 04-0787) and the (110), (200), (211), (112), (202), (222), (420), (402) planes of body-centered tetragonal (bct) Al_2_Cu intermetallic compound (JCPDS 25-0012), respectively. The optical micrograph of E-Al_82_Cu_18_ alloy sheets reveals that the eutectic solidification produces an ordered lamellar nanostructure of alternating α-Al and intermetallic Al_2_Cu lamellas with thicknesses of ~150 nm and ~270 nm (Fig. 1e and Supplementary Fig. 1), i.e., the lamellar spacing of ~420 nm. This microstructure is also illustrated by scanning electron microscope (SEM) backscattered electron image and its corresponding energy dispersive spectroscopy (EDS) elemental mapping of Al and Cu. As shown in Fig. 1f, both Al and Cu atoms periodically distribute in the E-Al_82_Cu_18_ alloy, depending on the presence of alternating Al and Al_2_Cu nanolamellas. Figure 1g shows a high-resolution transmission electron microscope (HRTEM) image of Al/Al_2_Cu interfacial region, viewed along their <111> and <10$\bar{2}$> zone axis. In view of the phase separation triggered by eutectic reaction^40,41^, there present distinctly isolated monometallic Al and intermetallic Al_2_Cu regions, which are identified by their fast Fourier transform (FFT) patterns of fcc and bct crystallographic structures (Fig. 1h, i). Owing to the high oxophilicity of Al^10,11,22–25,34,35^, it is reasonable to observe thin amorphous oxide shell with a thickness of ~4 nm on the constituent α-Al lamellas of the E-Al_82_Cu_18_ alloy (Fig. 1j, k). Nevertheless, X-ray photoelectron spectroscopy (XPS) measurements indicate that in addition to the chemical state of Al^3+^ due to the formation of Al_2_O_3_ layer, the Al and Cu components at the surface layer of E-Al_82_Cu_18_ alloy are primarily in the metallic states because of the conductive Al_2_Cu lamellas (Supplementary Fig. 2a, b), which not only facilitate electron transfer through the amorphous Al_2_O_3_ surface layer but pair with their neighboring Al lamellas to form localized Al/Al_2_Cu galvanic couples in charge/discharge processes^36,37,42^.

### Electrochemical characterizations of the Al–Cu alloys

**Fig. 2:**
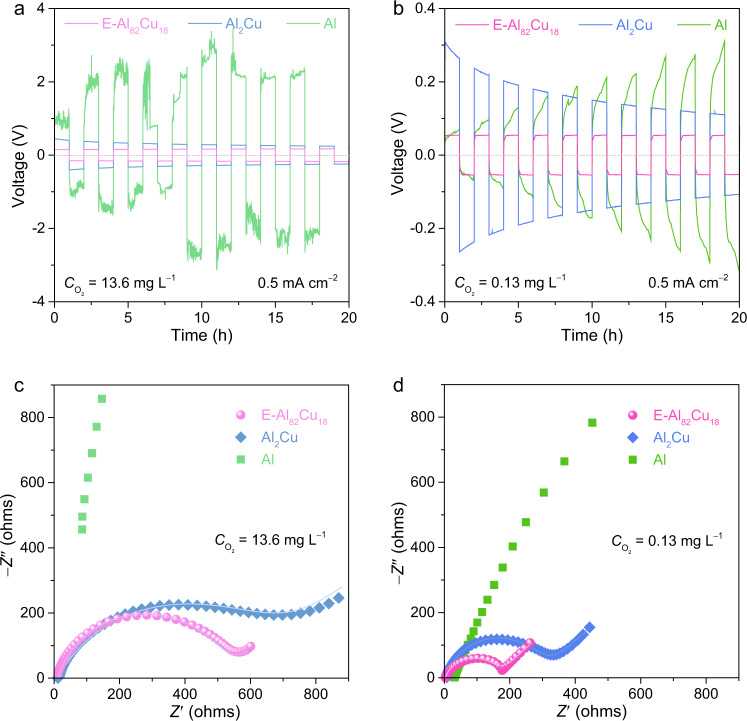
Dependence of Al plating/stripping behaviors of eutectic Al-Cu alloys on oxygen concentrations. **a**, **b** Al stripping/plating voltage profiles of E-Al_82_Cu_18_ (pink line), Al_2_Cu (blue line), and pure Al (green line) electrodes in their as-assembled symmetric cells in 2 M Al(OTF)_3_ aqueous electrolyte with *C*_O2_ = 13.6 (**a**) and 0.13 mg L^−1^ (**b**), which are purged by O_2_ and N_2_ for 2 h, respectively. Current density: 0.5 mA cm^−2^. **c**, **d** EIS spectra of as-assembled E-Al_82_Cu_18_, Al_2_Cu, and pure Al symmetric cells in 2 M Al(OTF)_3_ aqueous electrolyte with *C*_O2_ = 13.6 (**c**) and 0.13 mg L^−1^ (**d**). The symbols are the raw data of E-Al_82_Cu_18_ (pink spheres), Al_2_Cu (blue diamonds), and pure Al (green squares) symmetric cells while the lines represent the fit data of E-Al_82_Cu_18_ (pink line) and Al_2_Cu (blue line).

To investigate the influence of passivating oxide layer on the Al stripping/plating behaviors of Al-based electrodes, electrochemical measurements are carried out in symmetric cell configuration using 2 M Al(OTF)_3_ aqueous electrolytes with various oxygen concentrations (*C*_O2_), which are adjusted by purging O_2_ or N_2_ for different time (Supplementary Table 2). Figure 2a shows a representative voltage profile of symmetric E-Al_82_Cu_18_ cell during the Al stripping/plating at the current density of 0.5 mA cm^−2^, compared with those of symmetric Al_2_Cu and Al ones, in the O_2_-purged Al(OTF)_3_ aqueous electrolyte with *C*_O2_ = 13.6 mg L^−1^. The E-Al_82_Cu_18_ symmetric cell exhibits relative flat and symmetric voltage plateaus at Al stripping/plating steps despite the hysteresis voltage gradually increasing to ~180 mV from the initial 150 mV probably due to the continual formation of passivating oxide in such high-oxygen-concentration electrolyte (Supplementary Fig. 3a). This is in sharp contrast with the monometallic Al symmetric cell, of which the unstable overpotential runs up to as high as ~2000–3000 mV due to side reactions such as hydrogen evolution reaction and Al oxidation reaction (Fig. 2a and Supplementary Fig. 3b)^11,14^. While for the Al_2_Cu symmetric cell, it takes initial high overpotential of ~400 mV to strip Al from thermodynamically stable intermetallic Al_2_Cu phase. As the stripped Al fully takes part in the subsequent stripping/plating cycles, the overpotential gradually decreases to ~195 mV (Fig. 2a and Supplementary Fig. 3c), which is comparable to the value of E-Al_82_Cu_18_ symmetric cell because of the formation of additional Al/Al_2_Cu galvanic couples^36,37,42^.

While in the Al(OTF)_3_ aqueous electrolyte with a low oxygen concentration, these Al-based electrodes have their surface oxidation to be alleviated for improved Al stripping/plating (Supplementary Fig. 3a–c). As shown in Supplementary Fig. 3d, the overpotentials of these Al-based symmetric cells evidently decrease as the *C*_O2_ is reduced to 0.13 mg L^−1^. Figure 2b compares the initial voltage profiles of E-Al_82_Cu_18_, Al_2_Cu, and Al symmetric cells during the Al stripping/plating at 0.5 mA cm^−2^, in the N_2_-purged Al(OTF)_3_ aqueous electrolyte with *C*_O2_ = 0.13 mg L^−1^. As a consequence of notably suppressing the production of additional oxide, the E-Al_82_Cu_18_ symmetric cell has the stable voltage plateaus of as low as ~53 mV, only one sixth of the initial overpotentials (~300 mV) that are taken to strip Al from the intermetallic Al_2_Cu matrix for subsequent Al stripping/plating cycling in the Al_2_Cu symmetric cells. The less polarization of E-Al_82_Cu_18_ cell is probably due to the lamellar nanostructure of E-Al_82_Cu_18_ electrode, in which the constituent metallic α-Al and intermetallic Al_2_Cu lamellas play distinct roles in the Al stripping/plating cycles. By virtue of their different corrosion potentials^36,37,42^, the less-noble α-Al thermodynamically prefers to work as the electroactive material to supply Al^3+^ charge carriers, and the more-noble Al_2_Cu pairs with the constituent α-Al to form localized galvanic couples to trigger the Al stripping and serves as 2D nanopattern to guide the subsequent Al plating. No matter in which electrolyte with the *C*_O2_ from 13.6 to 0.13 mg L^−1^, the lamellar nanostructure improves the Al stripping/plating behaviors of E-Al_82_Cu_18_ (Supplementary Fig. 3a), compared with the monometallic Al that as a hostless electrode undergoes an increasing polarization process due to uncontrollable Al stripping/plating and unavoidable hydrogen evolution and Al oxidation reactions (Supplementary Fig. 3b)^11,23^. Their different Al stripping/plating behaviors are further investigated by using cyclic voltammetry (CV) in the N_2_-purged Al(OTF)_3_ aqueous electrolyte with *C*_O2_ = 0.13 mg L^−1^, where the E-Al_82_Cu_18_, Al_2_Cu, and Al materials are used as the working and counter electrodes and the Al wire as the reference electrode in a three-electrode cell configuration. As shown in Supplementary Fig. 4, the E-Al_82_Cu_18_ electrode exhibits improved symmetric Al stripping/plating behaviors, with an onset potential of as low as 0 V versus Al/Al^3+^ and an improved current density compared to the other Al-based electrodes. This is in sharp contrast to the intermetallic Al_2_Cu with strong Cu–Al covalent bonds and the monometallic Al with native oxide layer, which have their onset potentials of Al stripping to reach ~96 and ~172 mV, respectively, along the low current densities. The Al/Al_2_Cu galvanic couple enhanced Al stripping/plating kinetics is also demonstrated by electrochemical impedance spectroscopy (EIS) measurements of symmetric E-Al_82_Cu_18_, Al_2_Cu, and Al cells (Supplementary Fig. 5a–c). Figure 2c, d show the representative Nyquist plots, comparing the EIS spectra of all Al-based symmetric cells in the O_2_- and N_2_-purged Al(OTF)_3_ aqueous electrolytes with *C*_O2_ = 13.6 and 0.13 mg L^−1^, respectively. Therein, the E-Al_82_Cu_18_ symmetric cells display characteristic semicircles in the high- and middle-frequency range and inclined lines at the low frequencies, in contrast to those of the Al_2_Cu and Al ones with much larger diameters of semicircles. At high frequencies, the intersection point on the real axis represents the intrinsic resistance of both electrolyte and electrode (*R*_I_). In the middle-frequency range, the diameter of the semicircle corresponds to the parallel connection of the charge transfer resistance (*R*_CT_) of Al stripping/plating and the constant phase element (CPE). The slope of the inclined line at low frequencies is the Warburg resistance (*Z*_w_). Based on these general descriptors in the equivalent circuit (Supplementary Fig. 5d), the EIS spectra are analyzed using the complex nonlinear least-squares fitting method. Supplementary Fig. 6a, b compare the *R*_I_ and *R*_CT_ values of all Al-based electrodes in the Al(OTF)_3_ aqueous electrolytes with different *C*_O2_, where the E-Al_82_Cu_18_ always has the lowest *R*_I_ and *R*_CT_ values. At *C*_O2_ = 0.13 mg L^−1^, the *R*_I_ of E-Al_82_Cu_18_ electrode is as low as ~3 Ω because there forms an ultrathin oxide layer to facilitate the Al stripping/plating. Triggered by the periodical Al/Al_2_Cu galvanic couples, the E-Al_82_Cu_18_ electrode has the *R*_CT_ of ~160 Ω, more than twenty-fold lower than that of the monometallic Al with a thicker passivating oxide layer (~3880 Ω) (Supplementary Table 3).

**Fig. 3:**
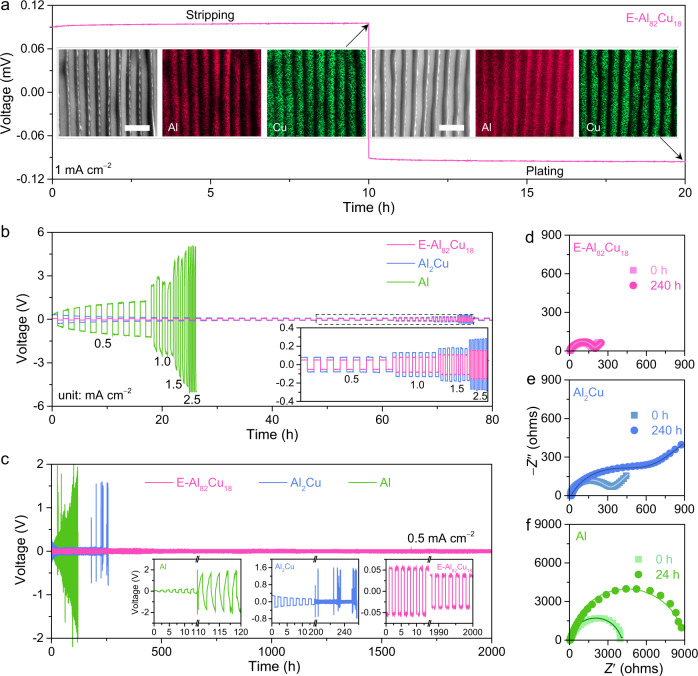
Electrochemical characterizations of the Al-based symmetric cells. **a** Typical stripping/plating voltage profile (pink line) of E-Al_82_Cu_18_ symmetric cells in 2 M Al(OTF)_3_ aqueous electrolyte with *C*_O2_ = 0.13 mg L^−1^. Current density: 1 mA cm^−2^. Insets: representative SEM images and the corresponding SEM-EDS elemental mappings of Al (in red) and Cu (in green) for the E-Al_82_Cu_18_ electrode after Al stripping (left) and then Al plating processes (right). Scale bars, 1 μm. **b** Comparison of rate performance for symmetric cells of E-Al_82_Cu_18_ (pink line), Al_2_Cu (blue line), and Al (green line) electrodes in 2 M Al(OTF)_3_ aqueous electrolyte with *C*_O2_ = 0.13 mg L^−1^ at various current densities of 0.5, 1.0, 1.5, 2.5 mA cm^−2^. Inset: enlarged voltage-time profiles comparing the stripping/plating behaviors of E-Al_82_Cu_18_ (pink line) and Al_2_Cu (blue line) electrodes at different current densities. **c** Long-term cycling stability of Al stripping/plating for symmetric cells based on E-Al_82_Cu_18_ (pink line), Al_2_Cu (blue line), and Al (green line) electrodes at 0.5 mA cm^−2^ in 2 M Al(OTF)_3_ aqueous electrolyte with *C*_O2_ = 0.13 mg L^−1^. Inset: voltage evolutions for Al (left), Al_2_Cu (middle), and E-Al_82_Cu_18_ (right). **d**–**f** EIS spectra of E-Al_82_Cu_18_ (**d**), Al_2_Cu (**e**), and Al (**f**) symmetric cells before and after the stripping/plating cycling measurements for 240 h, 240 h, and 24 h, respectively, in 2 M Al(OTF)_3_ aqueous electrolyte with *C*_O2_ = 0.13 mg L^−1^. The square and circle symbols are the raw data of E-Al_82_Cu_18_ (**d**), Al_2_Cu (**e**), and Al (**f**) symmetric cells before and after Al stripping/plating for 240 h, respectively, in which the lines represent their fit data.

To identify the specific roles of α-Al and Al_2_Cu nanolamellas in the E-Al_82_Cu_18_ electrodes, ex-situ SEM-EDS elemental mapping characterization is conducted after deep Al stripping and plating at 1 mA cm^−2^ for 10 h in the Al(OTF)_3_ aqueous electrolyte with *C*_O2_ = 0.13 mg L^−1^ (Fig. 3a). As shown in a typical SEM backscattered electron image of the Al-stripped E-Al_82_Cu_18_ electrode (left inset of Fig. 3a), the constituent α-Al lamellas as electroactive materials selectively dissolve during the Al stripping process while the intermetallic Al_2_Cu ones are left to form a lamella-nanostructured 2D pattern. This is also illustrated by its corresponding SEM-EDS elemental mapping of Al and Cu (left insets of Fig. 3a), wherein the Al atoms distribute along the Cu-rich Al_2_Cu lamellas. During the subsequent Al electroplating process, the Al is incorporated into the stripped E-Al_82_Cu_18_ along the in-situ formed structural bidimentional Al_2_Cu nanopatterns. As shown in the SEM-EDS elemental mapping images of Al-stripped and -plated E-Al_82_Cu_18_ (right insets of Fig. 3a), the electrodeposited Al atoms uniformly distribute in the channels sandwiched between the Al_2_Cu lamellas, the same as the pristine E-Al_82_Cu_18_ (Fig. 1e). According to the voltage profiles of Al stripping/plating processes, the energy efficiency (EE) is evaluated to be ~99.4% in terms of the equation ${{{{{\rm{EE}}}}}}=\int I{V}_{{{{{{\mathrm{stripping}}}}}}}(t){{{{{\mathrm{d}}}}}}t/\int I{V}_{{{{{{\mathrm{plating}}}}}}}(t){{{{{\mathrm{d}}}}}}t$, indicating the high Al reversibility of E-Al_82_Cu_18_ electrode. Here *I* is the current density, *V*_stripping_(*t*) *and V*_plating_(*t*) are the stripping and plating voltages at the time (*t*).

Owing to the lamella-nanostructured Al_2_Cu pattern that enhances the Al stripping/plating kinetics of the constituent α-Al lamellas, the symmetric E-Al_82_Cu_18_ cell exhibits a better rate performance in the aqueous Al(OTF)_3_ electrolyte with *C*_O2_ = 0.13 mg L^−1^. As shown in Fig. 3b, the E-Al_82_Cu_18_ symmetric cell has a steadily increasing hysteresis of ~31, ~56, and ~103 mV when the current density is increased from 0.5 to 1.0, 1.5, and 2.5 mA cm^−2^. These hysteresis voltages are much lower than the values of the symmetric cells based on intermetallic Al_2_Cu (~51, ~95, and ~192 mV) and monometallic Al (~1750, ~2990, and ~4530 mV) electrodes. Figure 3c compares the Al stripping/plating cycling stabilities of all Al-based symmetric cells. Obviously, the voltage profile of E-Al_82_Cu_18_ symmetric cell does not display evident fluctuation in the long-term cycling at 0.5 mA cm^−2^ for more than 2000 h, except for the slight reduction in overpotential from initial ~53 mV to final ~37 mV probably due to the formation of less and less oxide (right inset of Fig. 3c) and the negligible hydrogen evolution (Supplementary Fig. 7a). This is in contrast with those of Al_2_Cu and Al symmetric cells with much larger voltage hysteresis and fluctuation at 180 h and 26 h, respectively (Fig. 3c). When extending the cycling time, there take place severe side reactions of hydrogen evolution and Al oxidation along with the Al stripping/plating processes, especially in the monometallic Al symmetric cell (left inset of Fig. 3c and Supplementary Fig. 7b). The hydrogen generation is identified by in-situ gas chromatography (Supplementary Fig. 7c). The hydrogen production increases the pH value of electrolytes to facilitate the oxidation of Al metal and thus aggravate side reactions^11,43^, which leads to cell case damage and electrolyte leak (Supplementary Fig. 8). As attested by the more intensive Raman bands and the change of chemical states of Al in XPS spectra (Supplementary Figs. 9 and 10), there indeed produces additional Al_2_O_3_ on the monometallic Al electrode after 40 stripping/plating cycles. While in the E-Al_82_Cu_18_ symmetric cell, the surface oxide of E-Al_82_Cu_18_ electrode is probably below the detection limit for the Raman spectroscopy measurements (Supplementary Figs. 11 and 12), which enables highly reversible Al stripping/plating at low overpotential. Furthermore, there does not observe any bubbles on the E-Al_82_Cu_18_ electrodes during the Al stripping/plating processes (Supplementary Fig. 7b). The improved cycling stability of E-Al_82_Cu_18_ electrode is also justified by the unconspicuous change of EIS spectra during the Al stripping/plating processes (Fig. 3d). Relative to the initial values of *R*_I_ and *R*_CT_, they only increase by ~2 and ~20 Ω after 120 cycles, respectively, much lower than those of intermetallic Al_2_Cu electrodes (~8 and ~290 Ω) (Fig. 3e and Supplementary Table 4). While the monometallic Al symmetric cell has its *R*_I_ and *R*_CT_ values to increase to ~36 and ~8855 Ω only after 12 cycles (Fig. 3f and Supplementary Table 4). By virtue of the high reversibility of Al stripping/plating, the E-Al_82_Cu_18_ electrode still keeps the initial lamella nanostructure even after more than 1000 cycles (2000 h) (Supplementary Fig. 13a), in stark contrast to the Al_2_Cu and Al electrodes that are performed for only 125 and 20 cycles of Al stripping/plating, respectively. As shown in Supplementary Fig. 13b, c, there appear a large number of cracks on Al_2_Cu and Al electrodes. All these electrochemical and structural features verify the effective Al stripping/plating behaviors of E-Al_82_Cu_18_ electrode because of its lamellar nanostructure of alternating intermetallic Al_2_Cu and α-Al lamellas.

### Electrochemical energy storage performances of Al-ion full cells

To develop E-Al_82_Cu_18_-based AR-AMB full cells for practical use, a cathodic material of Al^3+^ pre-intercalated manganese oxide (Al_*x*_MnO_2_·*n*H_2_O) is prepared by a modified hydrothermal method. Supplementary Figure 14a, b show low-magnification SEM and TEM images of as-prepared Al_*x*_MnO_2_·*n*H_2_O, displaying a hierarchical nanostructure consisting of nanosheets with thickness of ~10 nm. The HRTEM image of Al_*x*_MnO_2_·*n*H_2_O nanosheets illustrates the nature of layered crystalline structure (inset of Supplementary Fig. 14b). According to the spectral features of the Mn–O vibrations^44,45^, the characteristic Raman bands at 506, 573, and 641 cm^−1^ unveil a birnessite-type structure (Supplementary Fig. 14c)^46^. This is further confirmed by the obvious diffraction peaks in the XRD patterns of Al_*x*_MnO_2_·*n*H_2_O at 2θ = 10.9°, 25.2°, 36.7°, 65.9°, which correspond to the 001, 002, 110, and 020 reflections of birnessite (JCPDS 43–1456) (Supplementary Fig. 14d). The diffraction peaks deviating from their corresponding line patterns indicates the pre-intercalation of hydrated Al^3+^ cation. In terms of the 001 diffraction peak position, the interlayer spacing of Al_*x*_MnO_2_·*n*H_2_O nanosheets is evaluated to be 0.811 nm, in agreement with the observation in the HRTEM image (inset of Supplementary Fig. 14b). The XPS survey spectrum attests to the presence of Al, Mn, and O atoms in the as-prepared Al_*x*_MnO_2_·*n*H_2_O nanosheets (Supplementary Fig. 15a), where the *x* value is evaluated to be ~0.12 according to inductively coupled plasma optical emission spectroscopy (ICP-OES) analysis (Supplementary Table 5). In high-resolution Al 2*p* XPS spectrum (Supplementary Fig. 15b), the characteristic peak at the binding energy of 75.0 eV is attributed to the pre-intercalated Al^3+^ cations that are engaged into the MnO_6_ sheets to adjust the chemical states of Mn^3+^ and Mn^4+^ (Supplementary Fig. 15c)^12,15^. O 1*s* XPS analysis demonstrates that there mainly exist three oxygen-containing species, i.e., the O_2_^−^ in MnO_6_ lattice, the OH^−^ and the H_2_O, to correspond to the peaks at the binding energies of 529.8, 530.9, and 533.0 eV (Supplementary Fig. 15d)^7,47^. Therein, the latter is assigned to both crystal water and constitution water, which are identified by thermogravimetric analysis (TGA) at the temperature below 510 °C. As shown by the TGA profile (Supplementary Fig. 15e), the weight loss below 120 °C is attributed to the removal of the crystal water^48^. When increasing temperature from 120 °C to 510 °C, the corresponding weight loss is ascribed to the constitutional water due to the formation of hydrated Al^3+^ with a high enthalpy^49^.

**Fig. 4:**
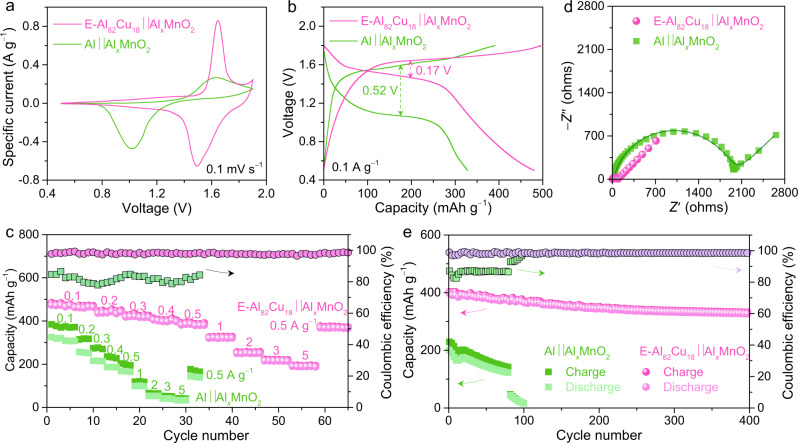
Electrochemical characterizations of the aqueous Al-ion full cells. **a** Representative CV curves for full E-Al_82_Cu_18_ | |Al_*x*_MnO_2_ (pink line) and Al | |Al_*x*_MnO_2_ (green line) Al-ion cells in 2 M Al(OTF)_3_ aqueous electrolyte with *C*_O2_ = 0.13 mg L^−1^. Scan rate: 0.1 mV s^−1^. **b** Typical voltage profiles of E-Al_82_Cu_18_ | |Al_*x*_MnO_2_ (pink line) and Al | |Al_*x*_MnO_2_ (green line) cells at the specific current of 0.1 A g^−1^. **c** Comparison of rate performance and Coulombic efficiency for E-Al_82_Cu_18_ | |Al_*x*_MnO_2_ (pink spheres) and Al | |Al_*x*_MnO_2_ cells (green squares), which are performed at various specific currents from 0.1 to 5 A g^−1^. **d** EIS spectra of E-Al_82_Cu_18_ | |Al_*x*_MnO_2_ and Al | |Al_*x*_MnO_2_ full cells. The pink sphere and green square symbols are the raw data of E-Al_82_Cu_18_ | |Al_*x*_MnO_2_ and Al | |Al_*x*_MnO_2_ full cells while the light pink and dark green lines represent their fit data, respectively. **e** Capacity retentions and Coulombic efficiencies of E-Al_82_Cu_18_ | |Al_*x*_MnO_2_ (pink spheres) and Al | |Al_*x*_MnO_2_ cells (green squares) in a long-term charge/discharge cycling measurement at 0.5 A g^−1^.

Figure 4a shows representative cyclic voltammetry (CV) curves of full AR-AMB cells that are assembled with the E-Al_82_Cu_18_ alloy or monometallic Al anode and the Al_*x*_MnO_2_·*n*H_2_O cathode, i.e., E-Al_82_Cu_18_ | |Al_*x*_MnO_2_ or Al | |Al_*x*_MnO_2_, in 2 M Al(OTF)_3_ aqueous electrolyte with *C*_O2_ = 0.13 mg L^−1^. Though both E-Al_82_Cu_18_ | |Al_*x*_MnO_2_ and Al | |Al_*x*_MnO_2_ AR-AMB cells have the same cathode material of Al_*x*_MnO_2_·*n*H_2_O nanosheets, they exhibit distinct voltammetric behaviors due to their different anodes, i.e., the lamella-nanostructured E-Al_82_Cu_18_ and the monometallic Al, indicating the significance of Al-based anodes in determining electrochemical performance of full AR-AMB cells. By virtue of the improved Al stripping/plating properties of E-Al_82_Cu_18_ enabling a fast reaction kinetics of Al^3+^ intercalation/deintercalation in the Al_*x*_MnO_2_·*n*H_2_O, the E-Al_82_Cu_18_ | |Al_*x*_MnO_2_ cell shows enhanced current density and positively shifted voltages of anodic/cathodic peaks relative to the Al | |Al_*x*_MnO_2_. At the scan rate of 0.1 mV s^−1^, the anodic and cathodic peaks of E-Al_82_Cu_18_ | |Al_*x*_MnO_2_ can reach ~1.647 and ~1.491 V, respectively, with the voltage difference of ~156 mV. Whereas the voltage difference of anodic and cathodic peaks increases to ~673 mV when increasing the scan rate to 3 mV s^−1^ (Supplementary Fig. 16a), it is still much smaller than that of Al | |Al_*x*_MnO_2_ cell at the scan rate of 0.2 mV s^−1^ (~863 mV) (Supplementary Fig. 16b). These observations indicate the improved rate capability of E-Al_82_Cu_18_ | |Al_*x*_MnO_2_ cell. As shown in Supplementary Fig. 16c, the E-Al_82_Cu_18_ | |Al_*x*_MnO_2_ cell can achieve a specific capacity of as high as ~478 mAh g^−1^ (based on the loading mass of Al_*x*_MnO_2_ in the cathode) at 0.1 mV s^−1^ and retains ~249 mAh g^−1^ at 3 mV s^−1^ (i.e., the discharge time of 467 s), even comparable to that of Al | |Al_*x*_MnO_2_ cell (262 mAh g^−1^) at 0.2 mV s^−1^ (7000 s).

Figure 4b and Supplementary Fig. 17a, b show the representative voltage profiles for the galvanostatic charge and discharge of E-Al_82_Cu_18_ | |Al_*x*_MnO_2_ and Al | |Al_*x*_MnO_2_ AR-AMB cells, with the voltage plateaus being consistent with their corresponding redox peaks in the CV curves due to the intercalation/de-intercalation of Al^3+^ via Al_*x*_MnO_2_·*n*H_2_O + 3(*y*-*x*)*e*^−^ + (*y*-*x*)Al^3+^ ↔ Al_*y*_MnO_2_·*n*H_2_O (Fig. 4a and Supplementary Fig. 16a, b)^12^, which is demonstrated by XPS analysis of Al_*x*_MnO_2_ cathode after the discharge and charge (Supplementary Figs. 18 and 19). As shown in Supplementary Fig. 18a, b for the Mn 2*p* and Al 2*p* of the discharged Al_*y*_MnO_2_, the intercalation of Al^3+^ leads to the *y* value of as high as 0.56, accompanied by the change of chemical state of Mn from Mn^3+^ and Mn^4+^ to Mn^2+^. As for the charged Al_*x*_MnO_2_, the content of Al decreases to *x* = ~11 due to the de-intercalation of Al^3+^, where the chemical state of Mn changes to Mn^3+^ and Mn^4+^ from Mn^2+^ (Supplementary Fig. 19a, b). In the charge or discharge state, the F and S contents are detected to be constant probably due to the physical adsorption of OTF ligands on the surface of Al_*x*_MnO_2_ (Supplementary Figs. 18d, e and 19d, e). Evidently, the use of E-Al_82_Cu_18_ alloy anode enlists the E-Al_82_Cu_18_ | |Al_*x*_MnO_2_ cell to exhibit a higher discharge plateau and smaller voltage polarization, giving rise to a dramatically improved energy efficiency. As manifested by the charge/discharge voltage difference (Δ*E*) at the specific current of 100 mA g^−1^ (~0.2 C)^50^, the Δ*E* decreases to 0.17 V of E-Al_82_Cu_18_ | |Al_*x*_MnO_2_ from 0.52 V of Al | |Al_*x*_MnO_2_. Furthermore, the discharge capacity of E-Al_82_Cu_18_ | |Al_*x*_MnO_2_ reaches as high as ~480 mAh g^−1^, ~1.5-fold of the Al | |Al_*x*_MnO_2_ (~328 mAh g^−1^). Even as the rate increases to 10 C (i.e., 5000 mA g^−1^), it still stores/delivers the capacities of ~194/~190 mAh g^−1^ in 6 min (Fig. 4c), with a high Coulombic efficiency of ~98% (Supplementary Fig. 20). In comparison, the charge/discharge capacities of Al | |Al_*x*_MnO_2_ decrease to ~42/~33 mAh g^−1^ (Fig. 4c), with a lower Coulombic efficiency of ~78% (Supplementary Fig. 20). As a result, the E-Al_82_Cu_18_ | |Al_*x*_MnO_2_ achieves the highest specific energy of ~672 Wh kg^−1^ (energy density of 815 Wh L^−1^ based on the volume of cathode) at 100 mA g^−1^ and retains ~212 Wh kg^−1^ at 5000 mA g^−1^ (Supplementary Fig. 21)^51^, comparable to representative LIBs (Supplementary Table 6). These electrochemical energy storage properties of E-Al_82_Cu_18_ | |Al_*x*_MnO_2_ cell are due to the improved Al stripping/plating kinetics of the lamella-nanostructured E-Al_82_Cu_18_. As demonstrated in EIS analysis (Fig. 4d and Supplementary Fig. 22a, b), the E-Al_82_Cu_18_ | |Al_*x*_MnO_2_ cell has its *R*_I_ and *R*_CT_ values to be ~18 Ω and ~1836 Ω smaller than those of Al | |Al_*x*_MnO_2_ (Supplementary Fig. 22c, d and Supplementary Table 7). Supplementary Figure 23 shows the self-discharge behavior of E-Al_82_Cu_18_ | |Al_*x*_MnO_2_ cell. Similar to the Al | |Al_*x*_MnO_2_, the Al_82_Cu_18_ | |Al_*x*_MnO_2_ has an evident voltage drop in the initial 10 h. Owing to the sluggish intercalation kinetics of Al^3+^ in the Al_*x*_MnO_2_, the E-Al_82_Cu_18_ | |Al_*x*_MnO_2_ displays a voltage plateau in the subsequent 190 h, with a low self-discharge rate of ~0.57 mV h^−1^. Moreover, the E-Al_82_Cu_18_ | |Al_*x*_MnO_2_ cell also exhibits an improved cycling stability when performed by the galvanostatic charge/discharge at 500 mA g^−1^ in the voltage window between 0.5 and 1.8 V (Supplementary Fig. 24). As shown in Fig. 4e, it retains ~83% of the initial capacity after 400 cycles, along with the Coulombic efficiency of ~99% (Supplementary Fig. 25). In sharp comparison, the Al | |Al_*x*_MnO_2_ cell undergoes fast capacity degradation as well as low Coulombic efficiency in tens of cycles probably due to the poor reversibility of monometallic Al (Fig. 4e and Supplementary Fig. 25). Along with the cell-level capacity of 66.7 mAh g^−1^ and specific energy of 90.2 Wh kg^−1^, which are evaluated according to the methodology of practical assessment for aluminum battery technologies^25^, our full E-Al_82_Cu_18_ | |Al_*x*_MnO_2_ cell outperforms state-of-the-art aluminum batteries (Supplementary Table 8).

## Discussion

In conclusion, we have demonstrated eutectic engineering as an effective strategy to develop highly reversible Al-based alloy anodes, typically lamella-nanostructured E-Al_82_Cu_18_, for high-performance aqueous rechargeable Al-ion batteries. Triggered by in-situ eutectic solidification reaction, the E-Al_82_Cu_18_ has an ordered lamellar nanostructure composed of alternating monometallic α-Al and intermetallic Al_2_Cu nanolamellas, which pair with each other to form periodically localized galvanic couples of Al/Al_2_Cu. By making use of their different corrosion potentials, the less-noble α-Al lamellas work as electroactive materials to supply Al^3+^ charge carriers while the more-noble Al_2_Cu lamellas serve as 2D nanopatterns to guide highly reversible Al stripping and plating at low overpotentials, particularly in N_2_-purged aqueous Al(OTF)_3_ electrolyte with ultralow oxygen concentration of 0.13 mg L^−1^. As a consequence, the E-Al_82_Cu_18_ electrodes exhibit exceptionally Al stripping/plating stability for more than 2000 h, along with low overpotentials and high energy efficiency. These outstanding electrochemical properties enlist full cells of E-Al_82_Cu_18_ | |Al_*x*_MnO_2_ to deliver specific energy of as high as ~670 Wh kg^−1^ or energy density of 815 Wh L^−1^ (based on the mass or volume of Al_*x*_MnO_2_ cathode) and retain 80% capacity for more than 400 cycles.

## Methods

### Preparation of eutectic Al-Cu alloy anodes and Al_x_MnO_2_ nanosheet cathode

The lamella-nanostructured eutectic Al_82_Cu_18_ alloy (E-Al_82_Cu_18_) ingots were firstly produced by arc melting pure Al (99.994%, Sinopharm Chemical Reagent Co. Ltd) and Cu (99.996%, Sinopharm Chemical Reagent Co. Ltd) metals in an argon atmosphere. During the furnace cooling assisted by circulating water, there takes place a eutectic solidification reaction to form a lamellar nanostructure. The as-prepared E-Al_82_Cu_18_ was cut into ~400-μm-thick sheets along the perpendicular direction of lamellar structure using a diamond wire saw cutting machine (STX-202A), followed by a 7000-mesh sandpaper polishing procedure for further microstructural characterizations and electrochemical measurements. The length and width of Al_82_Cu_18_ alloy are 20 mm and 10 mm, respectively. The Al_2_Cu intermetallic compound sheets with a thickness of ~400 μm were prepared by the same procedure. In comparison, the commercial Al foils were polished with a 7000-mesh sandpaper to remove surface oxide for use as Al electrode. The Al^3+^ preintercalated manganese oxide (Al_*x*_MnO_2_·*n*H_2_O) nanosheets were synthesized by a modified hydrothermal method. In a Teflon-lined steel, autoclave contains a mixture of 20 mM KMnO_4_, 20 mM NH_4_Cl, and 5 mM Al(NO_3_)_3_, the hydrothermal synthesis was performed at 150 °C for 24 h, with a magnetically stirring at a speed of 250 rpm. After washing in ultrapure water, the as-prepared Al_*x*_MnO_2_·*n*H_2_O nanosheets were mixed with super-P acetylene black as the conducting agent and poly (vinylidene difluoride) as the binder in a weight ratio of 70 : 20 : 10 and then pasted on stainless steel foil (~20 μm thick, Bary Metallic Co., Ltd) with the loading mass of 1.0 mg cm^−2^ for the use of cathode materials.

### Physicochemical characterizations

The electronic microstructures of E-Al_82_Cu_18_ and Al_2_Cu alloy sheets were characterized by a field-emission scanning electron microscope equipped with an X-ray energy-dispersive spectroscopy (JEOL, JSM-6700F, 8 kV) and a field-emission transmission electron microscope (JEOL, JEM-2100F, 200 kV). The metallographic microstructure of E-Al_82_Cu_18_ alloy was observed on a confocal laser scanning microscope (OLS3000, Olympus) after a chemical etching in a Keller solution. X-ray diffraction measurements of all specimens were performed on a D/max2500pc diffractometer with a Cu *K*α radiation. Raman spectra were measured on a micro-Raman spectrometer (Renishaw) at the laser power of 0.5 mW, in which the laser with a wavelength of 532 nm was equipped. X-ray photoelectron spectroscopy analysis was conducted on a Thermo ECSALAB 250 with an Al anode. Charging effects were compensated by shifting binding energies based on the adventitious C 1*s* peak (284.8 eV). O_2_ concentrations and Cu/Al ion concentrations in electrolytes were analyzed by portable DO meter (az8403) and inductively coupled plasma optical emission spectrometer (ICP-OES, Thermo electron), respectively.

### Electrochemical characterizations

Symmetric coin-type cells of E-Al_82_Cu_18_, Al_2_Cu, and Al were assembled with their two identical electrodes separated by glass fiber membranes (GFMs) with a pore diameter of 1.2 μm and thickness of 260 μm, in 0.25 mL 2 M Al(OTF)_3_ aqueous solutions with O_2_ concentrations from 0.13 to 13.6 mg L^−1^, at 25 ± 0.5 °C. Therein, the O_2_ concentrations in the electrolytes were adjusted by purging N_2_ for 2, 0.5, and 0 h, and O_2_ for 1 and 2 h, respectively. Electrochemical impendence spectroscopy (EIS) measurements were conducted on as-assembled symmetric cells of E-Al_82_Cu_18_, Al_2_Cu, and Al over a frequency range from 100 kHz to 10 mHz (71 points) in quasi-stationary potential at the amplitude of the sinusoidal voltage of 10 mV. The electrochemical Al stripping/plating behaviors were measured in as-assembled E-Al_82_Cu_18_, Al_2_Cu, and Al symmetric cells at various specific currents. To illustrate their electrochemical stabilities, Al stripping/plating and EIS measurements were performed on the same symmetric cells during their long-term Al stripping/plating cycles. Fresh full aqueous Al-ion coin cells were constructed with the E-Al_82_Cu_18_ or Al sheet as the anode, the stainless-steel foil supported Al_*x*_MnO_2_·*n*H_2_O as the cathode, the GFM as the separator, the 0.25 mL 2 M Al(OTF)_3_ aqueous solution containing 0.2 M Mn(OTF)_2_ and O_2_ concentration of 0.13 mg L^−1^ as the aqueous electrolyte, for measurements of CV, galvanostatic charge/discharge curves, EIS, durability, and self-discharge, respectively, at 25 ± 0.5 °C. All these electrochemical energy-storage tests were in an open environment, not in a climatic/environmental chamber. CV measurements were conducted on an electrochemical analyzer (Ivium Technology) in the voltage range of 0.5 and 1.9 V at scan rates from 0.1 to 3 mV s^−1^. Galvanostatic charge/discharge curves were collected at different specific currents to demonstrate their rate performance. EIS measurements were performed in the frequency ranges from 100 kHz to 10 mHz (71 points) in quasi-stationary potential at the amplitude of the sinusoidal voltage of 10 mV. The durability performance of full cells were evaluated by performing charge/discharge cycles at 500 mA g^−1^ (1 C). Self-discharge measurements were carried out by charging Al_82_Cu_18_ | |Al_*x*_MnO_2_ and Al | |Al_*x*_MnO_2_ full cells to 1.8 V, followed by open-circuit potential self-discharging for 200 h.

#### Statistics and reproducibility

Experiments were reproducible.

Figure 1e, the experiments were performed twice with similar results.

Figure 1f, the experiments were performed twice with similar results.

Figure 1g, the experiments were performed twice with similar results.

Figure 1j, the experiments were performed twice with similar results.

Figure 3a, the experiments were performed twice with similar results.

Supplementary Figure 13a–c, the experiments were performed twice with similar results.

Supplementary Figure 14a, b, the experiments were performed twice with similar results.

## Supplementary information

Supplementary Information

Peer Review File

## Data Availability

All data supporting this study and its findings within the article and its Supplementary Information are available from the corresponding authors upon reasonable request.
