# Supplementary material for: Aluminum-copper alloy anode materials for high-energy aqueous aluminum batteries
Source: Nat Commun. 2022 Jan 31;13:576. doi: 10.1038/s41467-022-28238-3 (PMC8803968; doi:10.1038/s41467-022-28238-3)
Supplement: Supplementary file 1 — Supplementary Information [file 41467_2022_28238_MOESM1_ESM.pdf]

Supplementary Information

for

Aluminum-copper alloy anode materials for high-energy aqueous aluminum batteries

Ran et al

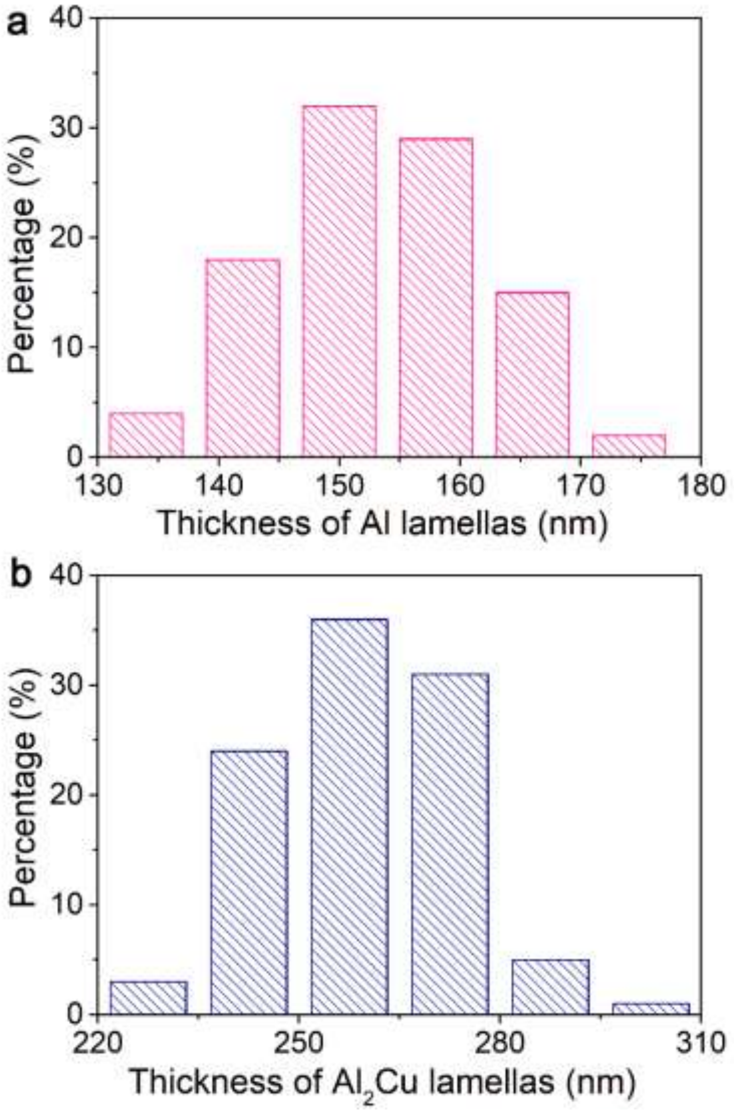

Supplementary Figure 1. Size distributions of Al and Al_2Cu lamellas in the E-$Al_{82}Cu_{18}$ alloy. a, Thickness of metallic α-Al lamellas. **b**, Thickness of intermetallic Al_2Cu lamellas.

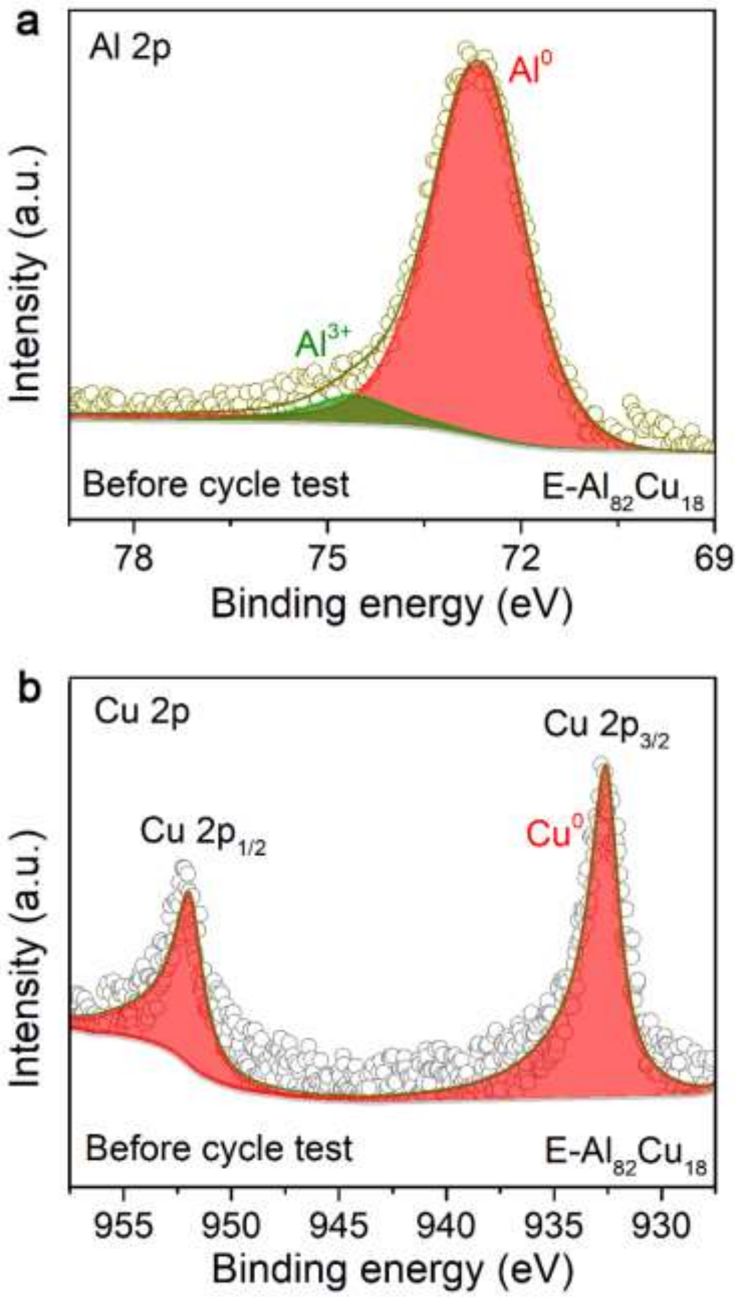

Supplementary Figure 2. XPS analysis of as-prepared E-$Al_{82}Cu_{18}$ alloy sheets. a, **b**, High-resolution XPS spectra of Al 2p (a) and Cu 2p (b) in as-prepared E-$Al_{82}Cu_{18}$ alloy.

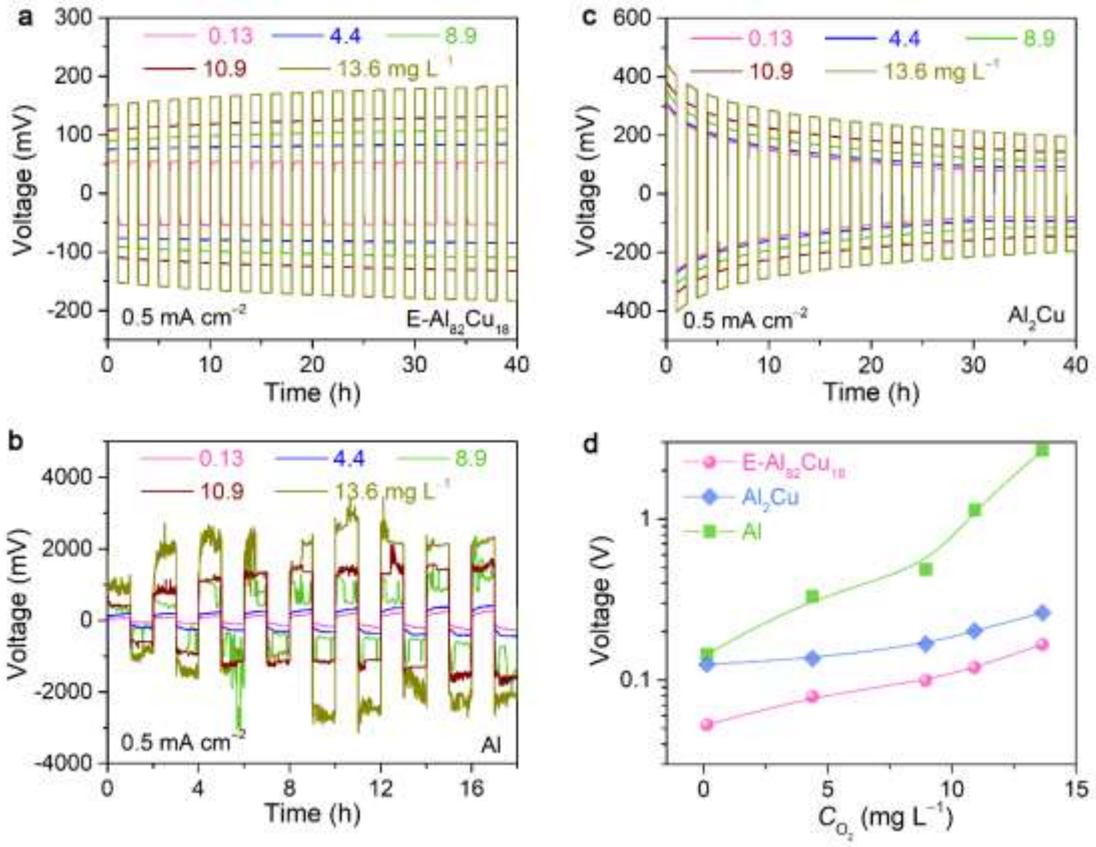

Supplementary Figure 3. Stripping/plating behaviors of Al-based symmetric cells in aqueous electrolytes with different O_2 concentrations. a-c, The stripping/plating behaviors of symmetric cells based on identical E-$Al_{82}Cu_{18}$ (a), Al (b) and Al_2Cu (c) electrodes, respectively, in 2 M $Al(OTF)_3$ aqueous electrolytes with O_2 concentrations of 0.13, 4.4, 8.9, 10.9, 13.6 mg L^{-1}. **d**, Hysteresis voltages of E-$Al_{82}Cu_{18}$, Al_2Cu and Al symmetric cells as a function of O_2 concentrations (C_{O2}).

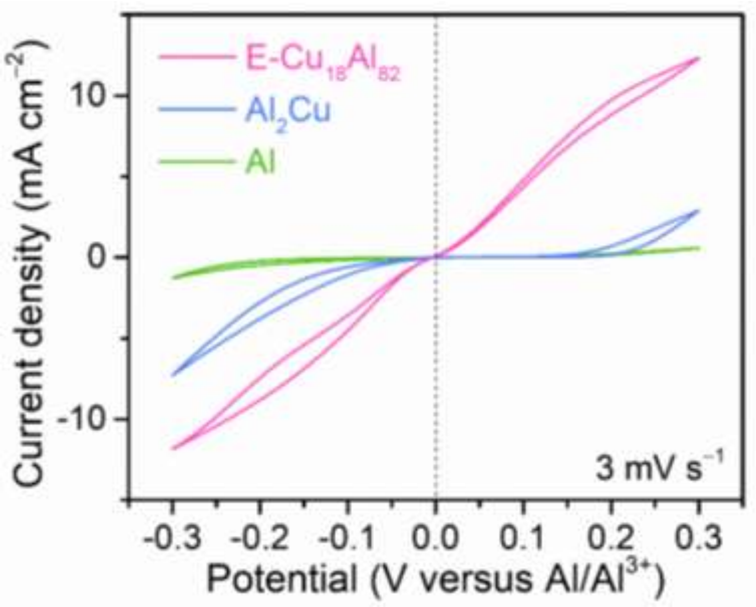

Supplementary Figure 4. Cyclic voltammogram of Al stripping/plating in three-electrode configuration cell, with E-$Al_{82}Cu_{18}$, Al_2Cu and Al sheets as the working and counter electrodes and Al wire as the reference electrode. The 1st CV curves of E-$Al_{82}Cu_{18}$, Al_2Cu and Al sheets during the Al stripping and plating. Electrolyte: in 2 M $Al(OTF)_3$ aqueous electrolytes with C_{O2} = 0.13 mg L^{-1}. Scan rate: 3 mV s^{-1}.

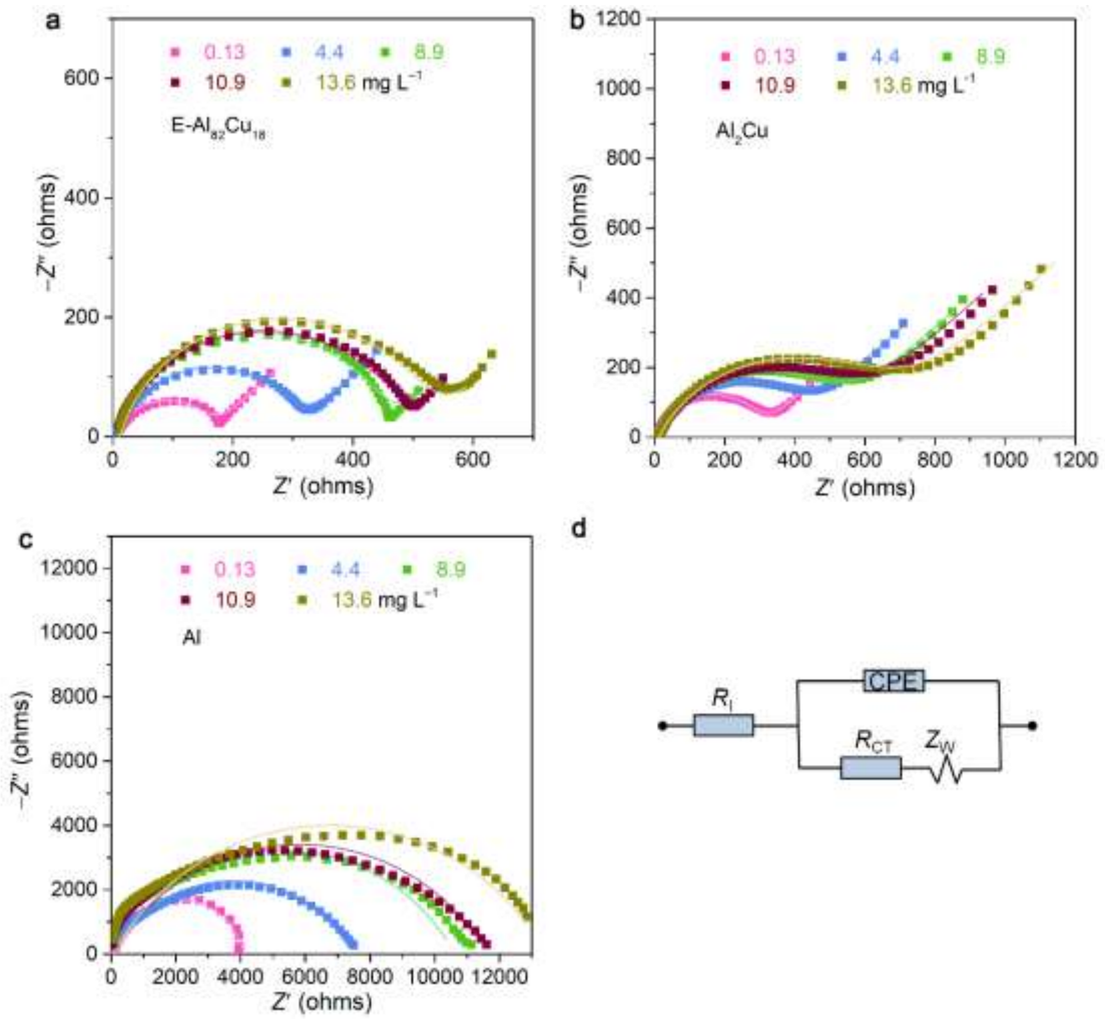

Supplementary Figure 5. EIS spectra of Al-based symmetric cells in aqueous electrolytes with different O_2 concentrations. a-c, The EIS spectra of as-assembled symmetric cells based on identical E-$Al_{82}Cu_{18}$ (a), Al_2Cu (b) and Al (c) electrodes, in 2 M $Al(OTF)_3$ aqueous electrolytes with O_2 concentrations of 0.13, 4.4, 8.9, 10.9, 13.6 mg L^{-1}. The square symbols in pink, blue, green, brown, dark yellow indicate the raw data for E-$Al_{82}Cu_{18}$ (a), Al_2Cu (b) and Al (c) symmetric cells in 0.13, 4.4, 8.9, 10.9, 13.6 mg L^{-1}, respectively, where the lines are their corresponding fit data. **d**, The equivalent circuit with the general descriptors: the intrinsic resistance of both electrolyte and electrode (R_I), the charge transfer resistance (R_{CT}), the constant phase element (CPE) and the slope of the inclined line at flow frequencies corresponding to the Warburg resistance (Z_W).

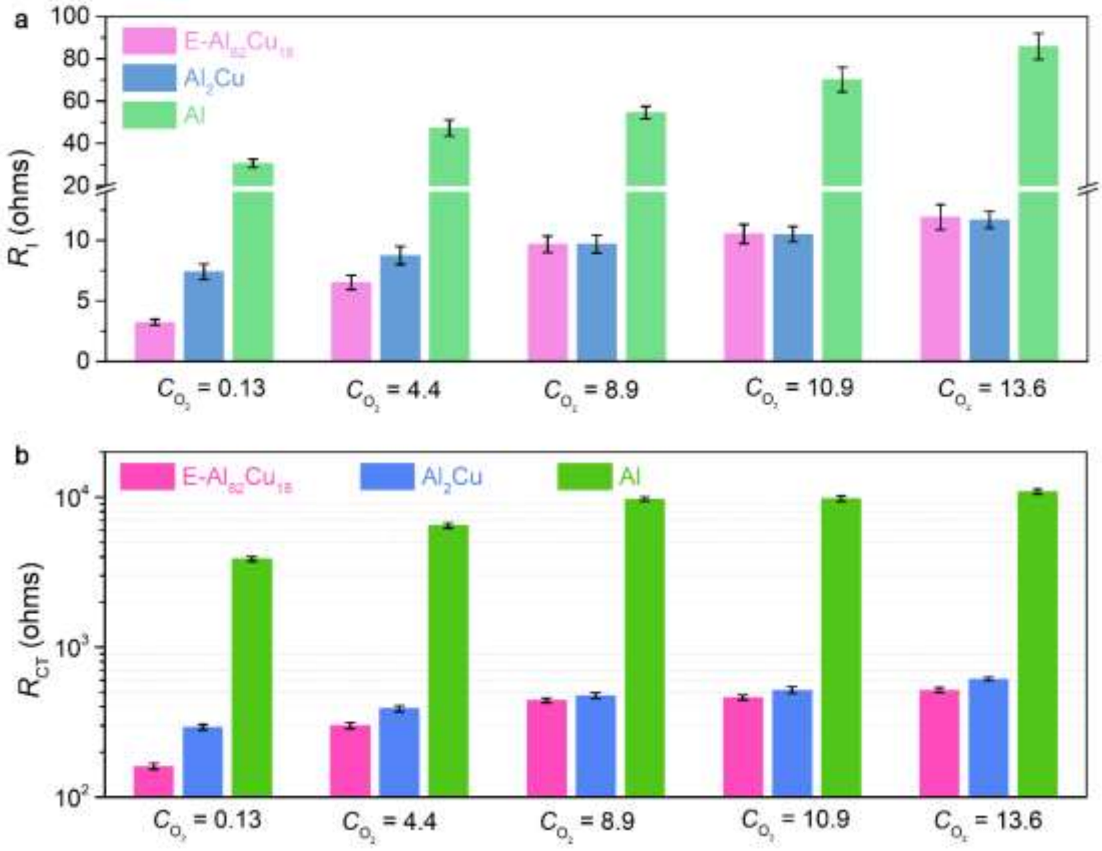

Supplementary Figure 6. Comparison of R_{CT} and R_I values of symmetric cells in aqueous electrolytes with different O_2 concentrations. a, The R_I values of E-$Al_{82}Cu_{18}$, Al_2Cu and Al electrodes based on symmetric cells in 2 M $Al(OTF)_3$ aqueous electrolytes with O_2 concentrations of 0.13, 4.4, 8.9, 10.9, 13.6 mg L^{-1}. **b**, The R_{CT} values of E-$Al_{82}Cu_{18}$, Al_2Cu and Al electrodes based on symmetric cells in 2 M $Al(OTF)_3$ aqueous electrolytes with O_2 concentrations of 0.13, 4.4, 8.9, 10.9, 13.6 mg L^{-1}. The values of R_I and R_{CT} for symmetric cells of E-$Al_{82}Cu_{18}$, Al_2Cu and Al electrodes are listed in Supplementary Table 3.

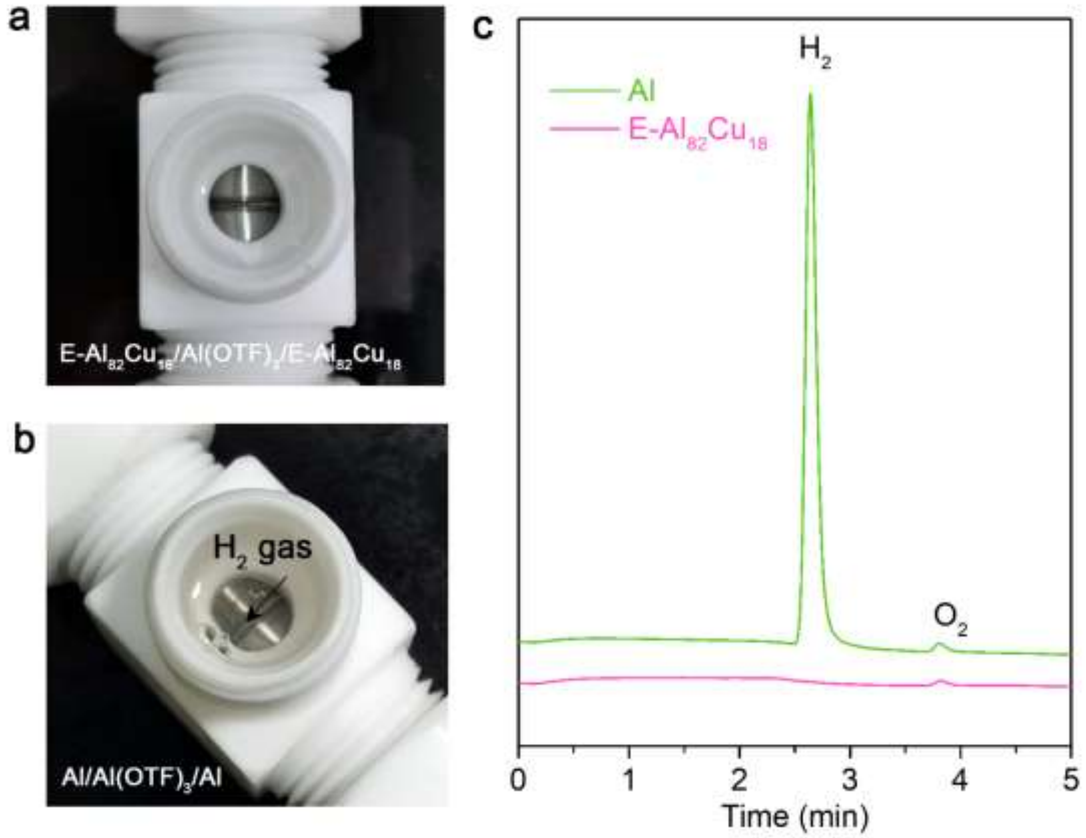

Supplementary Figure 7. Photograph images for symmetric cells constructed with E-Al$_{82}$Cu$_{18}$ and Al sheets, respectively, in Swagelok-type cells. a, Representative optical image of symmetric cell of E-Al$_{82}$Cu$_{18}$ during Al stripping/plating at 1 mA cm^{-2}, where there does not observe any bubbles. **b**, Representative optical image of symmetric cell of monometallic Al during Al stripping/plating at 1 mA cm^{-2}, where there generate obvious bubbles. **c**, Elution profiles of H_2 gases during the Al stripping/plating of monometallic Al and E-Al$_{82}$Cu$_{18}$ electrodes. Here the trace O_2 is probably due to the residual O_2 in the reactor.

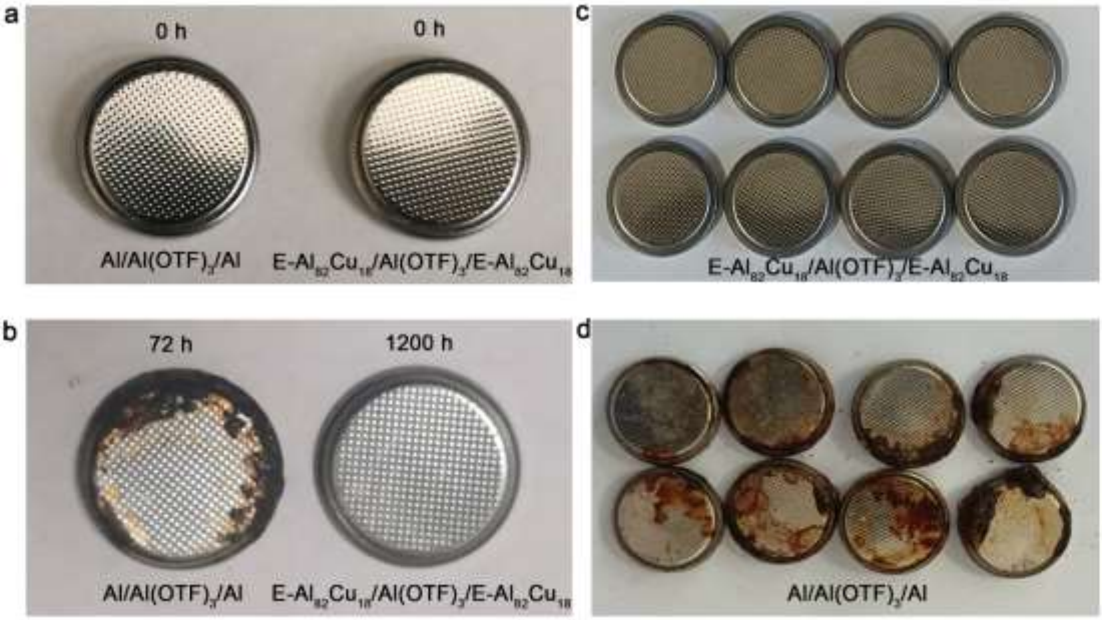

Supplementary Figure 8. Comparison of symmetric cells based on identical E-$Al_{82}Cu_{18}$ and Al electrodes before and after Al stripping/plating cycles in 2 M $Al(OTF)_3$ aqueous electrolyte with O_2 concentration of 0.13 mg L^{-1}. a, Optical photograph of E-$Al_{82}Cu_{18}$ (right) and Al (left) symmetric cells before Al stripping/plating cycles. **b**, Optical photograph of E-$Al_{82}Cu_{18}$ (right) and Al (left) symmetric cells before and after Al stripping/plating cycle measurements for 1200 h and 72 h, respectively. **c**, **d**, Optical photographs for many E-$Al_{82}Cu_{18}$ (c) and Al (d) symmetric cells after Al stripping/plating cycles.

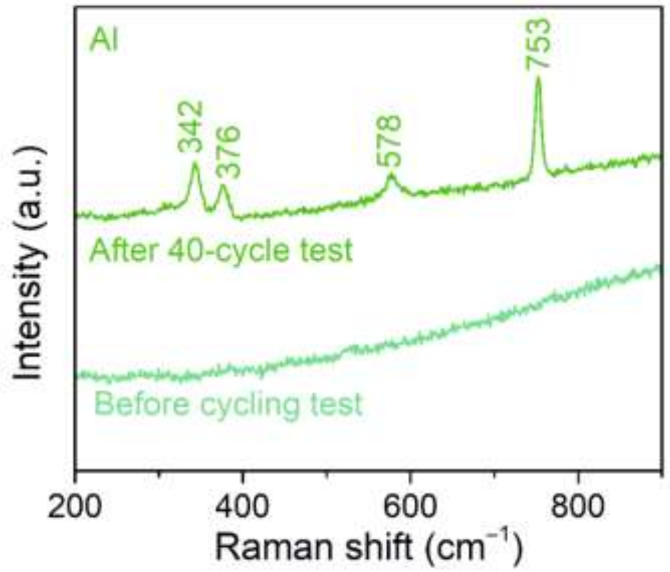

Supplementary Figure 9. Raman spectroscopy characterization of Al electrodes. Raman spectra of monometallic Al electrodes before and after Al stripping/plating cycles based on the Al symmetric cells in 2 M $Al(OTF)_3$ aqueous electrolyte with O_2 concentration of 0.13 mg L^{-1}. Here Raman bands at 376,578,753 cm^{-1} can be attributed to the E_g symmetric vibration mode of Al_2O_3. There does not observe the characteristic band corresponding to A_{1g} symmetric vibration mode due to the amorphous feature of Al_2O_3. The characteristic band at 342 cm^{-1} can be ascribed to the vibration of the Al-O bond in the presence of OH^-. The increase in intensity of Raman bands implies that there produces additional Al_2O_3 on monometallic Al electrode after Al stripping/plating cycles.

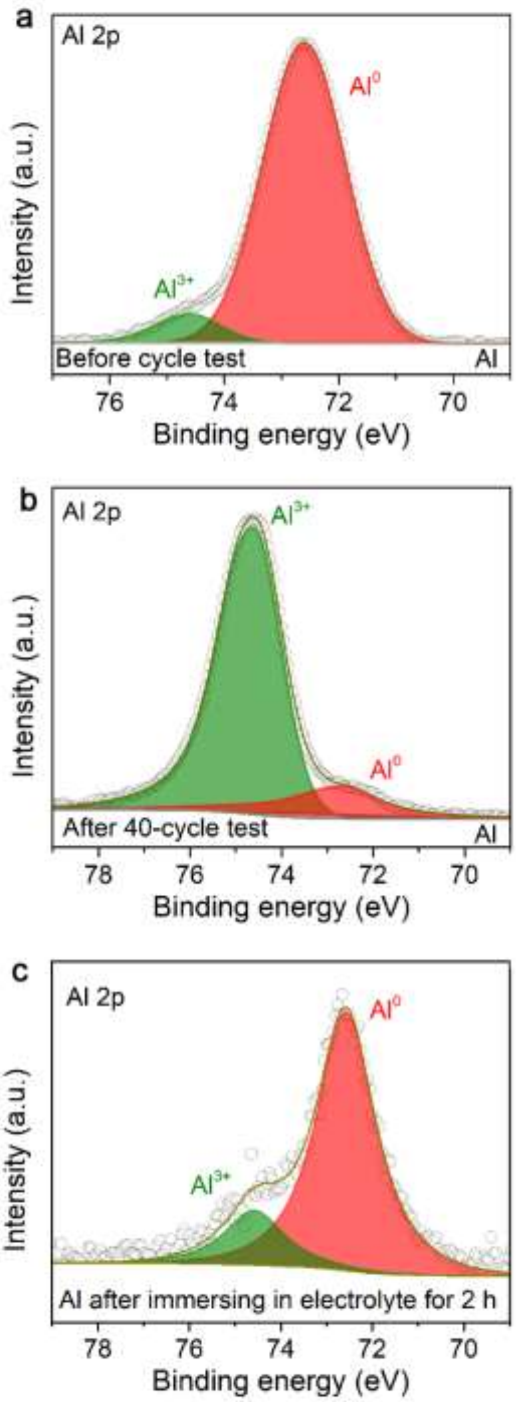

Supplementary Figure 10. Surface chemical states of Al electrodes before and after Al stripping/plating cycles in Al symmetric cells. a, **b**, Al 2p XPS spectra of monometallic Al electrode (a) before and (b) after 40 Al stripping/plating cycles in 2 M Al(OTF)$_3$ aqueous electrolyte with O_2 concentration of 0.13 mg L^{-1}. **c**, Al 2p XPS spectra of monometallic Al electrode after immersed in 2 M Al(OTF)$_3$ aqueous electrolyte for 2 h.

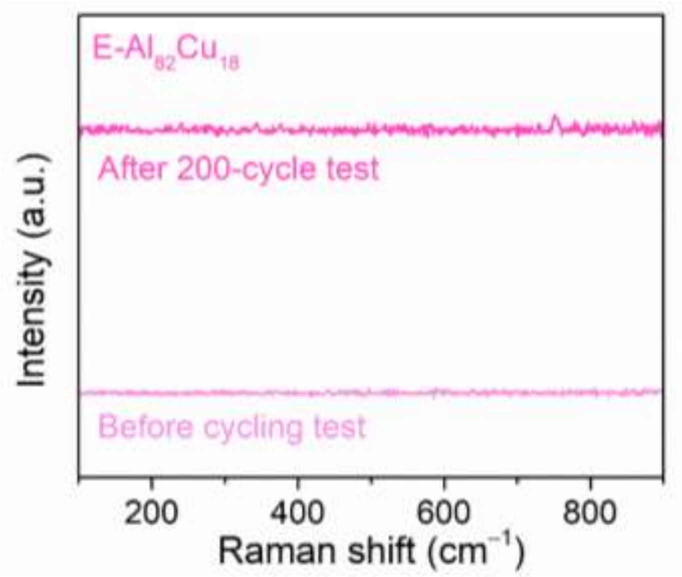

Supplementary Figure 11. Raman spectroscopy characterization of E-$Al_{82}Cu_{18}$ electrodes. Raman spectra of E-$Al_{82}Cu_{18}$ electrodes before and after Al stripping/plating for 200 cycles based on the E-$Al_{82}Cu_{18}$ symmetric cells in 2 M $Al(OTF)_3$ aqueous electrolyte with O_2 concentration of 0.13 mg L^{-1}.

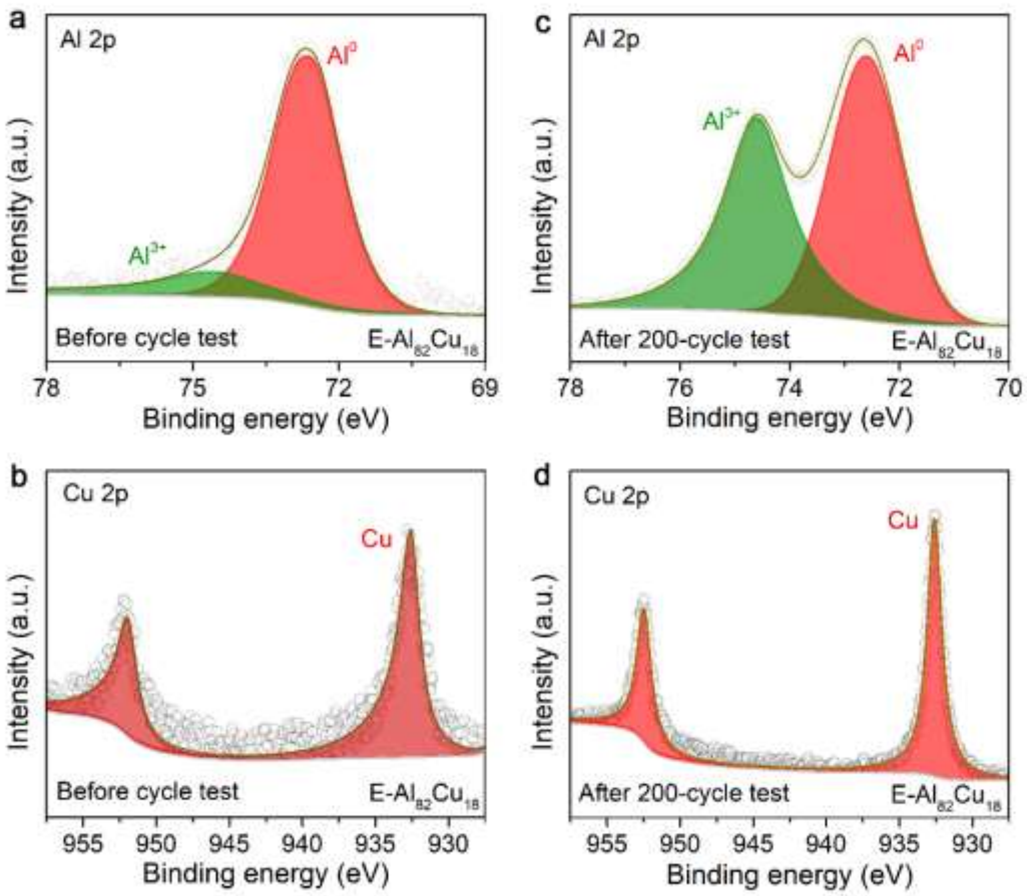

Supplementary Figure 12. Surface chemical states of Al and Cu in E-$Al_{82}Cu_{18}$ electrodes before and after Al stripping/plating cycles based on symmetric cells. a, **b**, Al 2p XPS spectra of Al in E-$Al_{82}Cu_{18}$ electrode (a) before and (b) after 200 Al stripping/plating cycles based on E-$Al_{82}Cu_{18}$ symmetric cells. **c**, **d**, Cu 2p XPS spectra of Cu in E-$Al_{82}Cu_{18}$ electrode (c) before and (d) after 200 Al stripping/plating cycles based on E-$Al_{82}Cu_{18}$ symmetric cells. Electrolyte: 2 M $Al(OTF)_3$ aqueous electrolyte, with O_2 concentration of 0.13 mg L^{-1}.

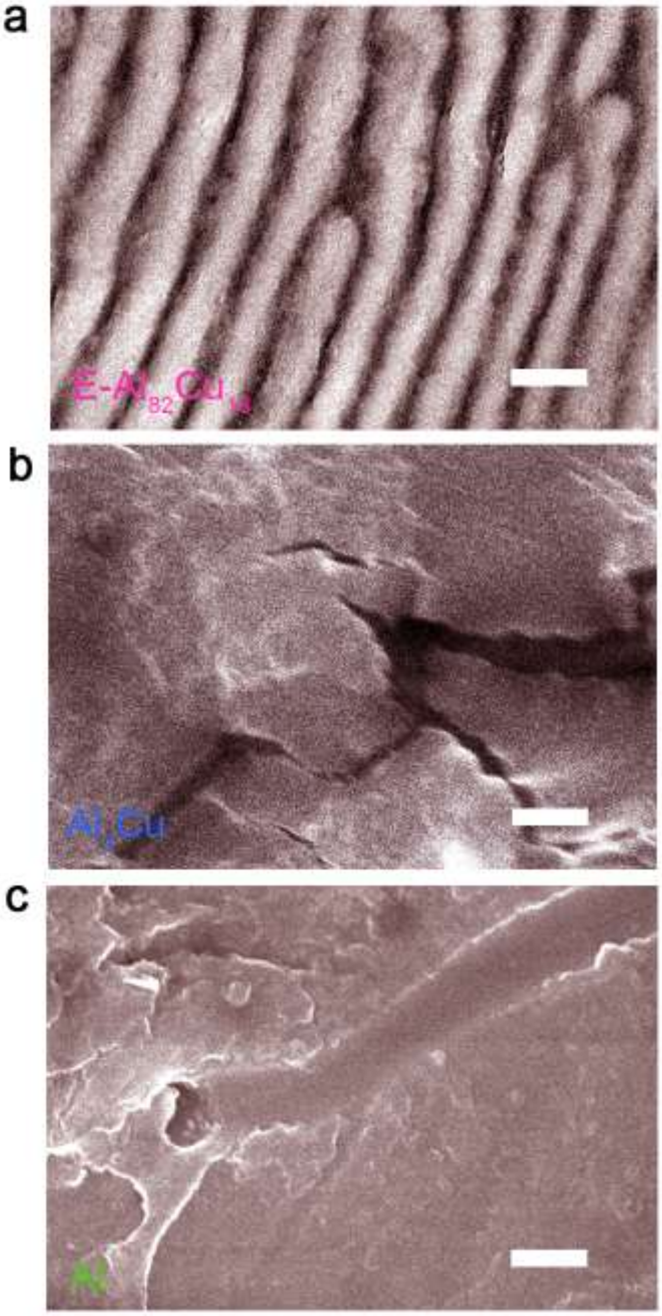

Supplementary Figure 13. SEM backscattered electron image of Al-based electrodes after Al stripping/plating cycling based on their symmetric cells in 2 M $Al(OTF)_3$ aqueous electrolyte with C_{O2} = 0.13 mg L^{-1}. a, SEM image of E-$Al_{82}Cu_{18}$ electrodes after the Al stripping/plating for 2000 h. Scale bars, 500 nm. **b**, SEM image of Al_2Cu electrodes after the Al stripping/plating for 250 h. Scale bar, 500 nm. **c**, SEM image of monometallic Al electrodes after the Al stripping/plating for 40 h. Scale bar, 500 nm.

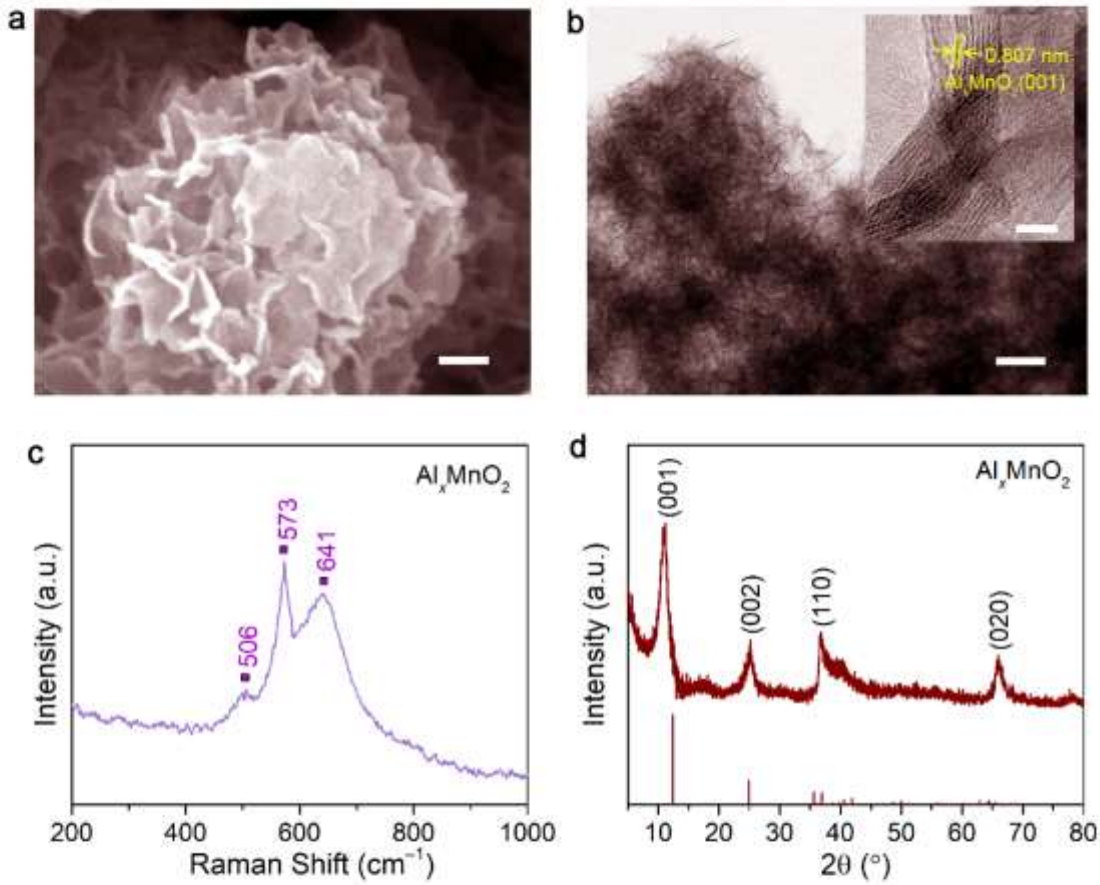

Supplementary Figure 14. Structure characterizations of Al_xMnO_2 nanosheets. a, Representative SEM image of Al_xMnO_2 nanosheets. Scale bar, 100 nm. **b**, Low-magnification TEM image of Al_xMnO_2 nanosheets. Scale bar, 100 nm. Inset: HRTEM image of Al_xMnO_2 nanosheets. Scale bar, 5 nm. **c**, Raman spectrum of as-prepared Al_xMnO_2 nanosheets. **d**, XRD patterns of as-prepared Al_xMnO_2 nanosheets. The line patterns show reference card 43-1456 for layered crystalline birnessite according to JCPDS.

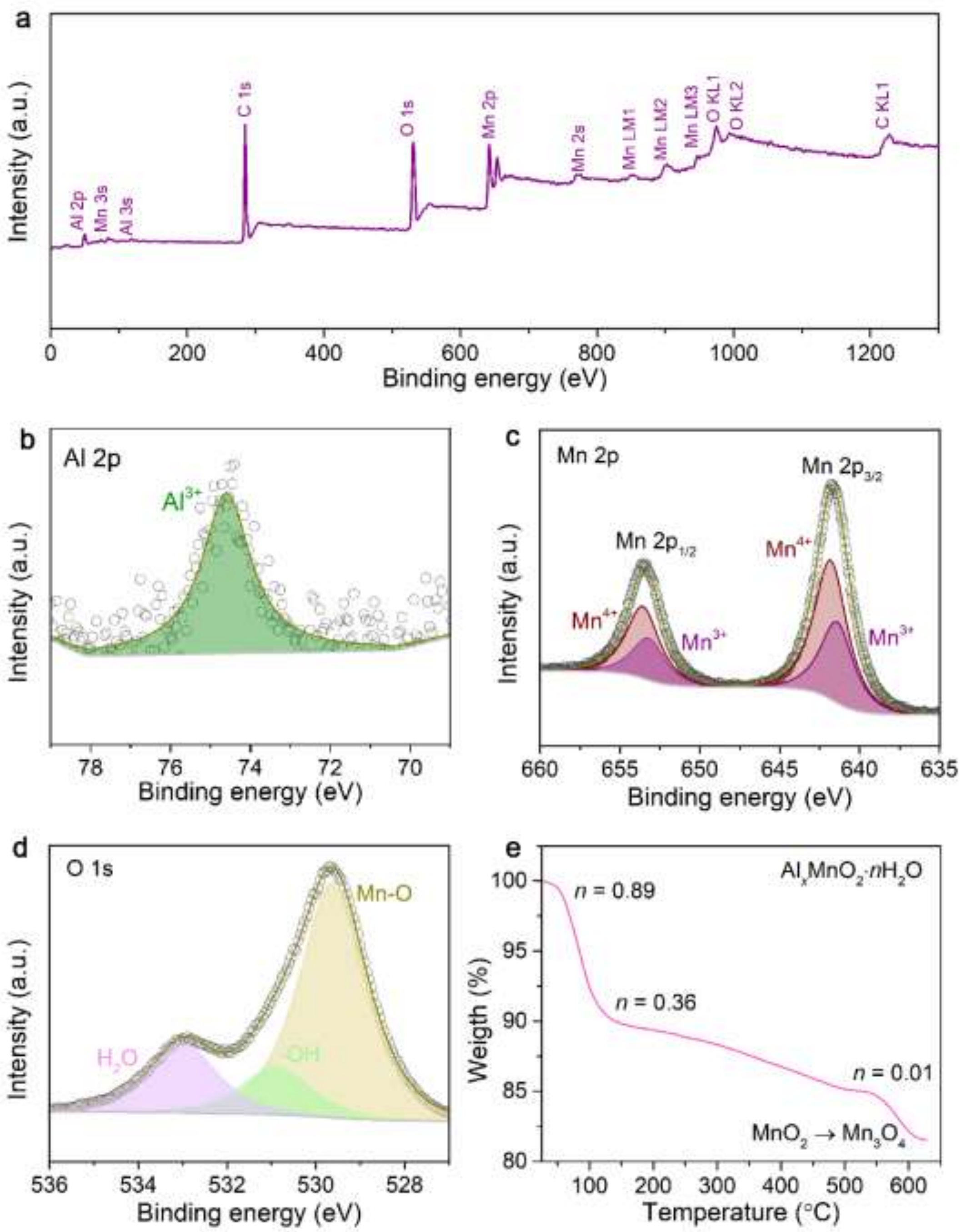

Supplementary Figure 15. XPS and TGA characterization of $Al_xMnO_2{\cdot}nH_2O$nanosheets. a, XPS survey spectrum of Al_xMnO_2. **b**, **c**, **d**, Al 2p (b) Mn 2p (c) and O 1s (d) XPS spectra of as-prepared Al_xMnO_2. **e**, TGA measurement reveals that ~0.89 mol of water exists in per mole of $Al_xMnO_2{\cdot}nH_2O$ at room temperature. The weight evolution is characterized by a steep loss between room temperature and 110 °C (removing crystal water, to $Al_xMnO_2{\cdot}0.36H_2O$), followed by a weight loss up to 530 °C (removing structure water, to $Al_xMnO_2{\cdot}0.01H_2O$).

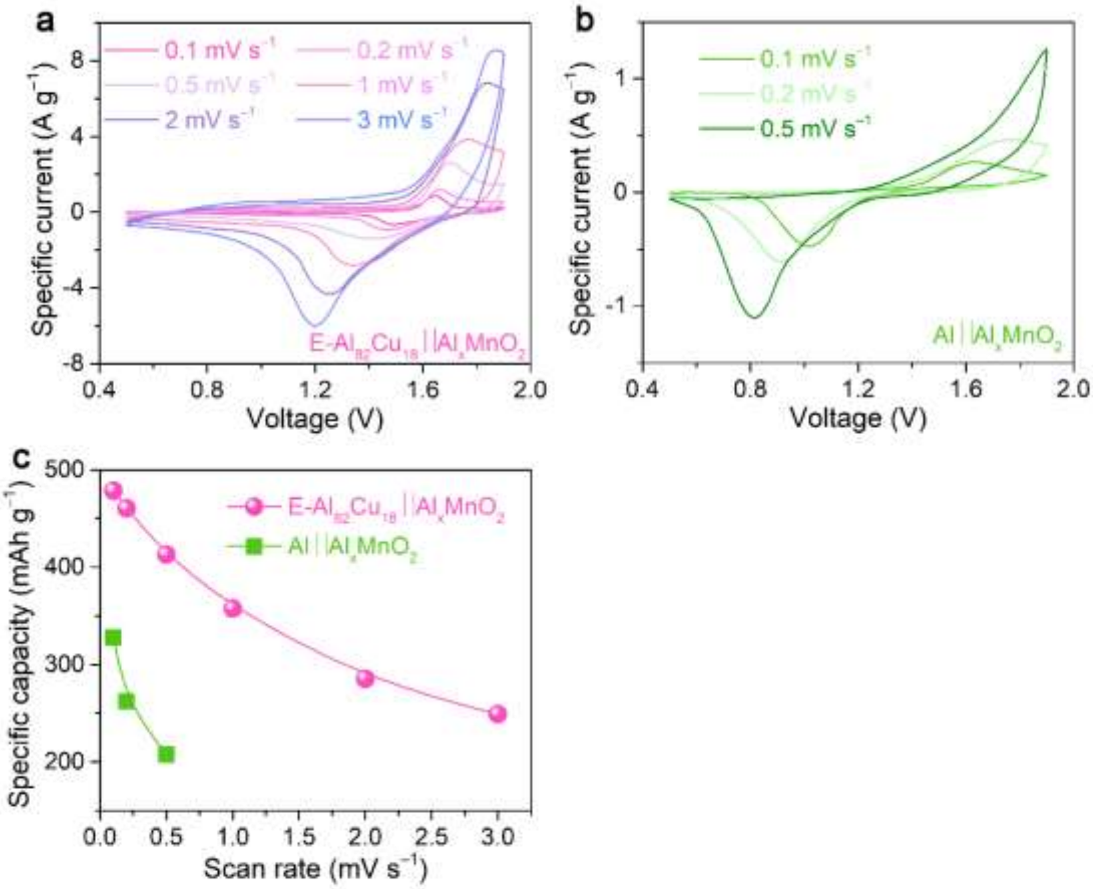

Supplementary Figure 16. Cyclic voltammetry behaviors of E-$Al_{82}Cu_{18}$||$Al_x MnO_2$ and Al||$Al_x MnO_2$ cells. a, Typical CV curves of E-$Al_{82}Cu_{18}$||$Al_x MnO_2$ cell at various scan rates from 0.1 to 3 mV s^{-1}. **b**, Typical CV curves of Al||$Al_x MnO_2$ cell at various scan rates from 0.1 to 0.5 mV s^{-1}. **c**, Specific capacities for E-$Al_{82}Cu_{18}$||$Al_x MnO_2$ and Al||$Al_x MnO_2$ cells at different scan rates.

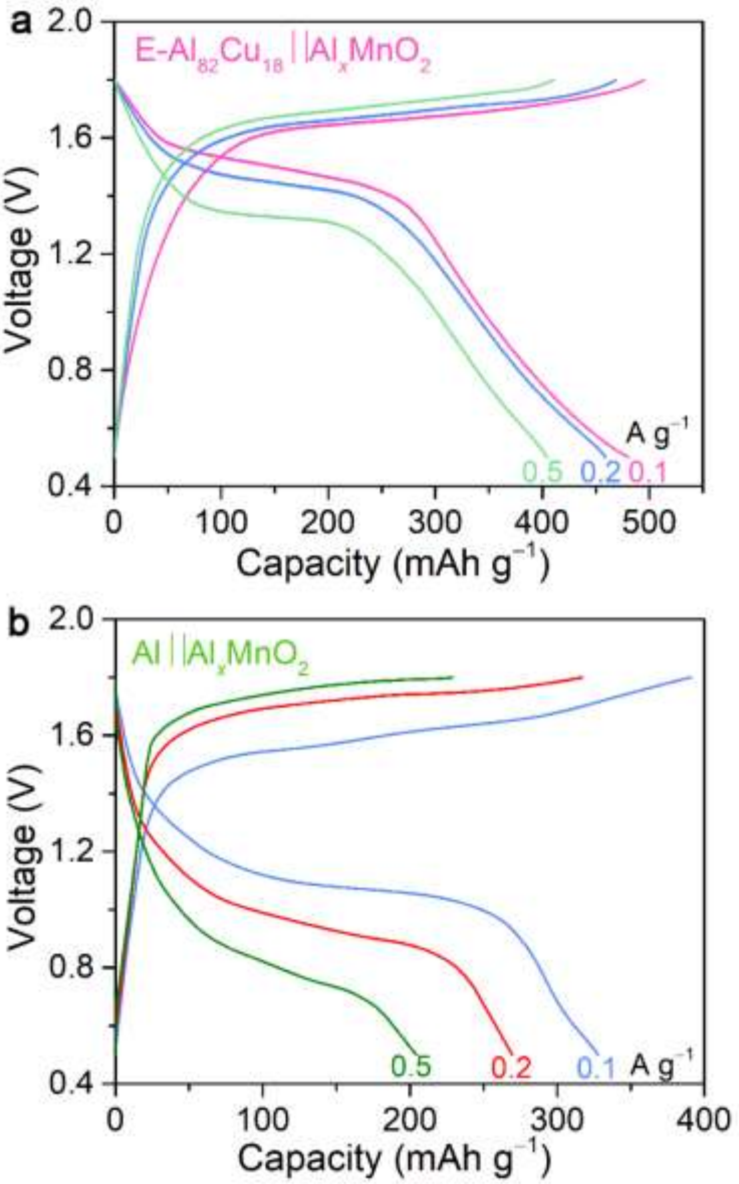

Supplementary Figure 17. Comparison of voltage profiles of $E\text{-}Al_{82}Cu_{18}||Al_xMnO_2$ and $Al||Al_xMnO_2$ cells. a, Typical voltage profiles of $E\text{-}Al_{82}Cu_{18}||Al_xMnO_2$ cell at galvanostatic charge/discharge with various specific currents from 0.1 to 0.5 A g^{-1}. **b**, Typical voltage profiles of $Al||Al_xMnO_2$ cell at galvanostatic charge/discharge with various specific currents from 0.1 to 0.5 A g^{-1}.

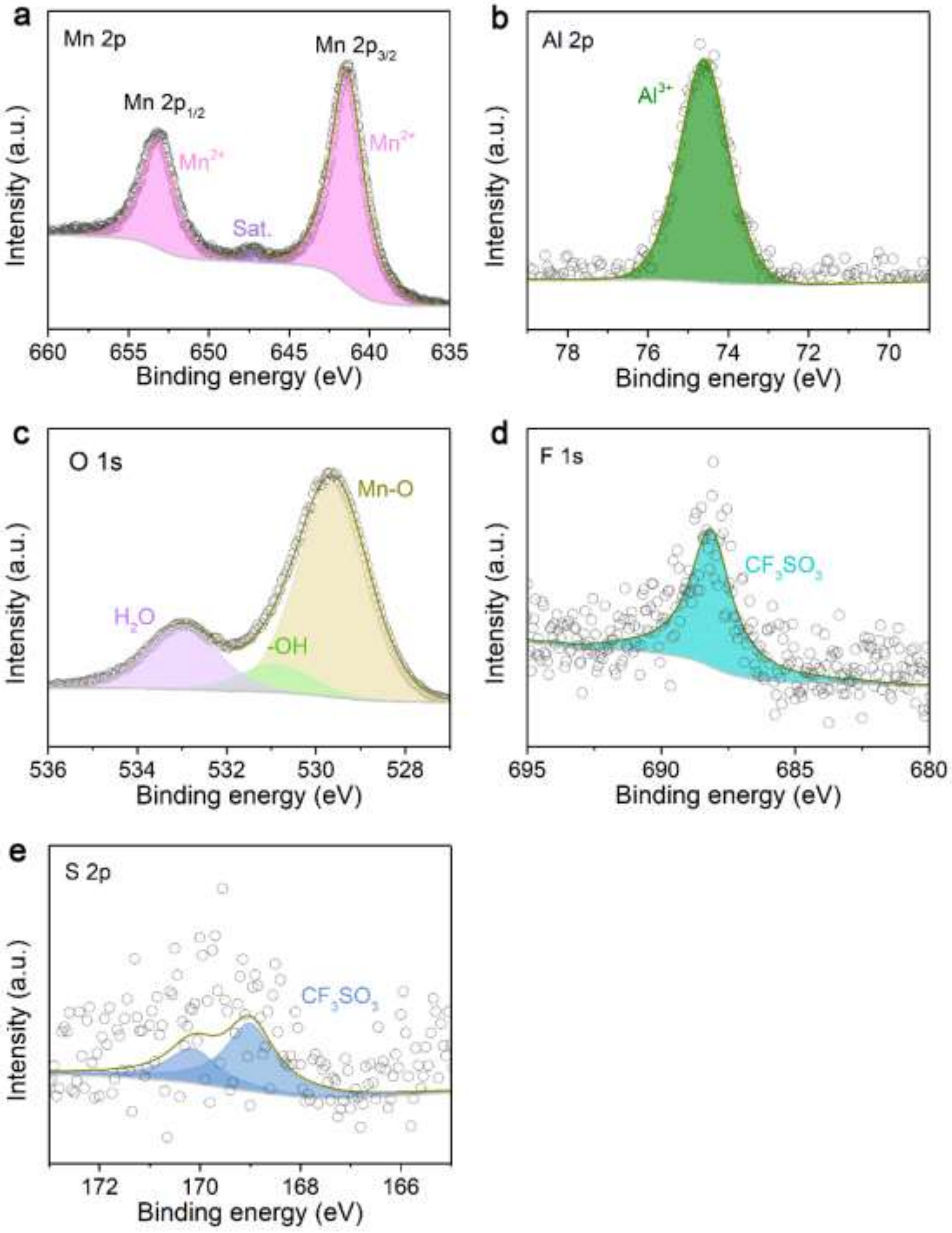

Supplementary Figure 18. XPS analysis of Al_xMnO_2 after discharging to 0.5 V. a, Mn 2p XPS spectrum. **b**, Al 2p XPS spectrum. **c**, O 1s XPS spectrum. **d**, F 1s XPS spectrum. **e**, S 2p XPS spectrum.

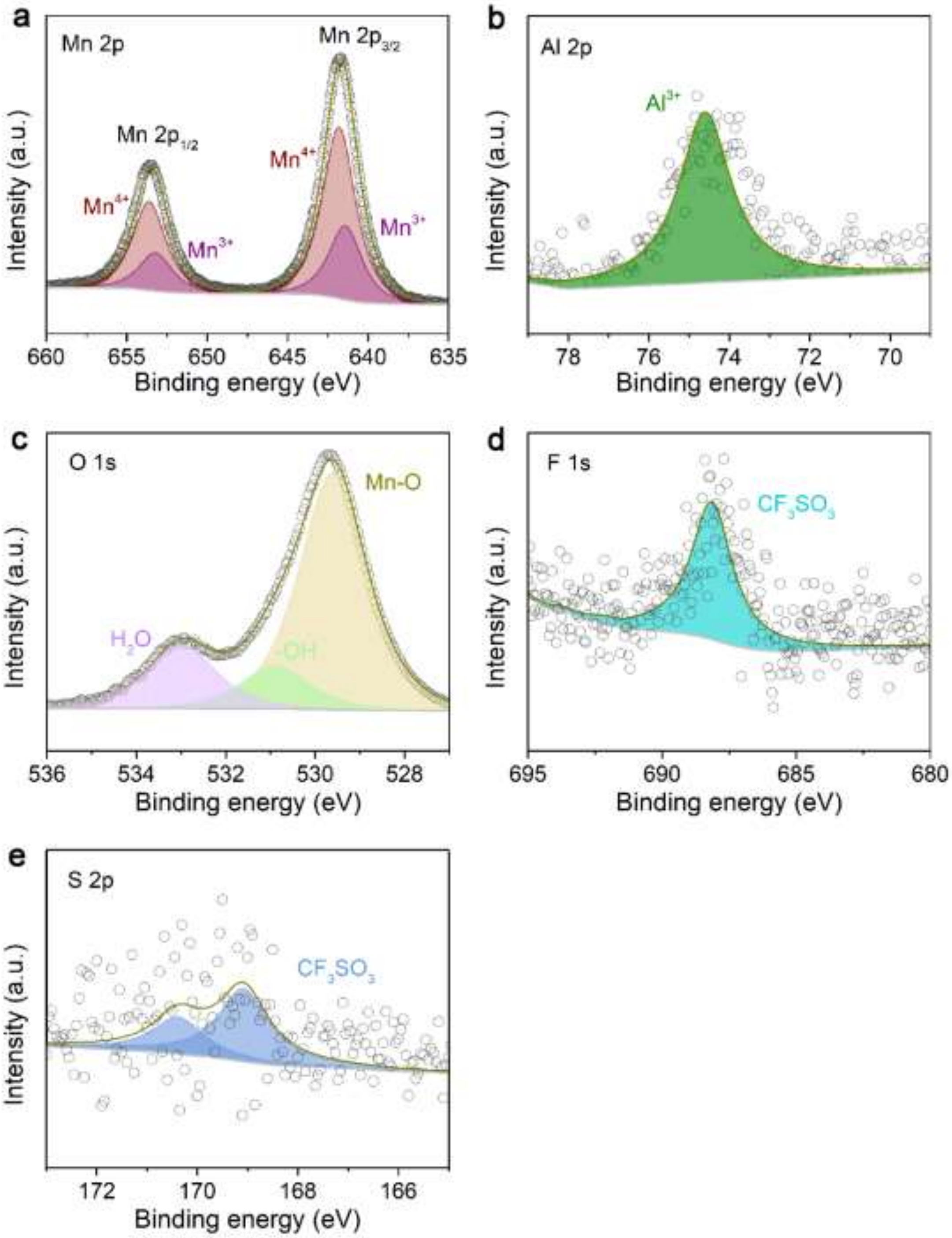

Supplementary Figure 19. XPS analysis of Al_xMnO_2 after charging to 1.8 V. a, Mn 2p XPS spectrum. **b**, Al 2p XPS spectrum. **c**, O 1s XPS spectrum. **d**, F 1s XPS spectrum. **e**, S 2p XPS spectrum.

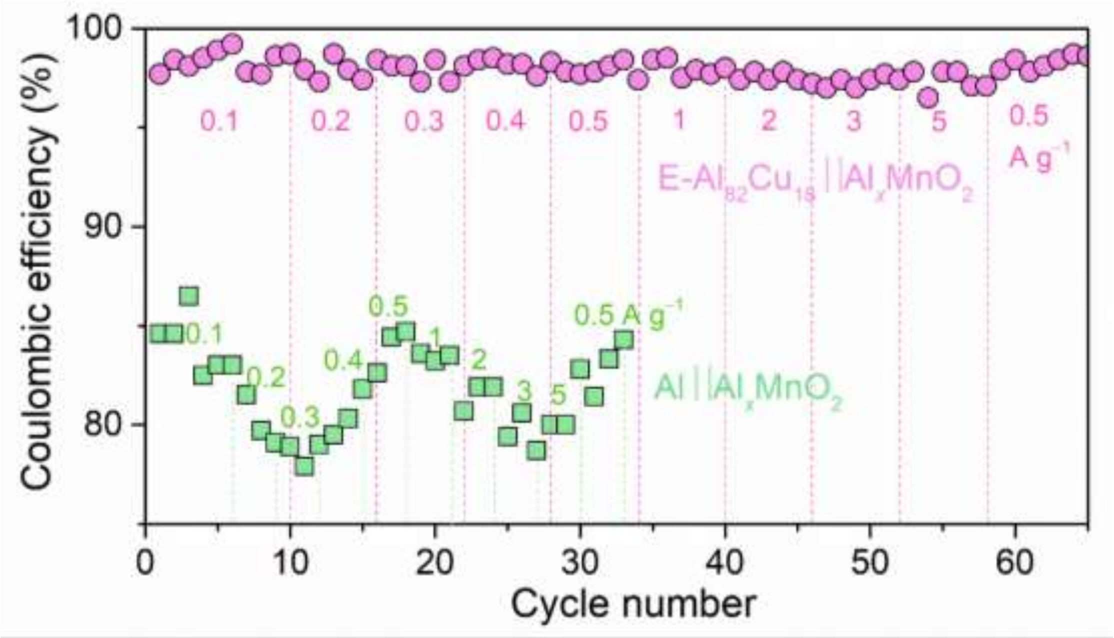

Supplementary Figure 20. Comparison of Coulombic efficiency for E-$Al_{82}Cu_{18}$||Al_xMnO_2 and Al||Al_xMnO_2 cells, which are performed at various specific currents from 0.1 to 5 A g^{-1}.

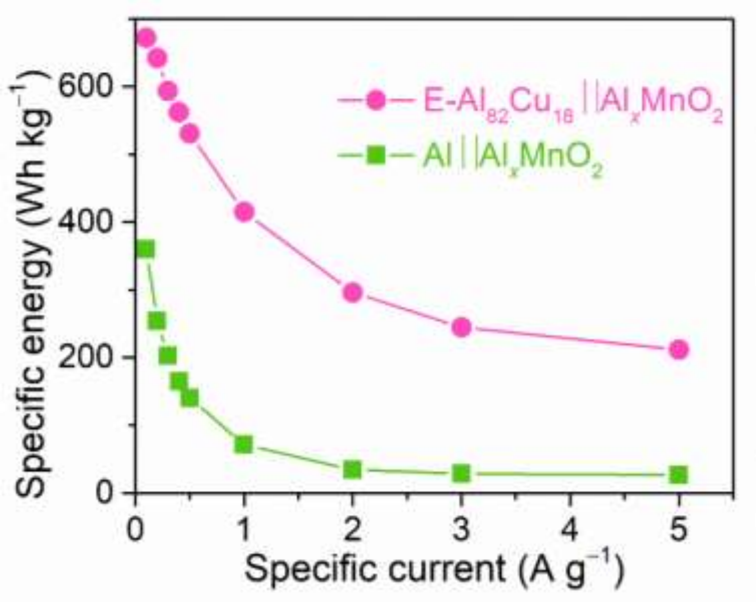

Supplementary Figure 21. The specific energy of E-$Al_{82}Cu_{18}$||Al_xMnO_2 and Al||Al_xMnO_2 full AR-AMB cells at various specific currents from 0.1 to 5 A g^{-1}.

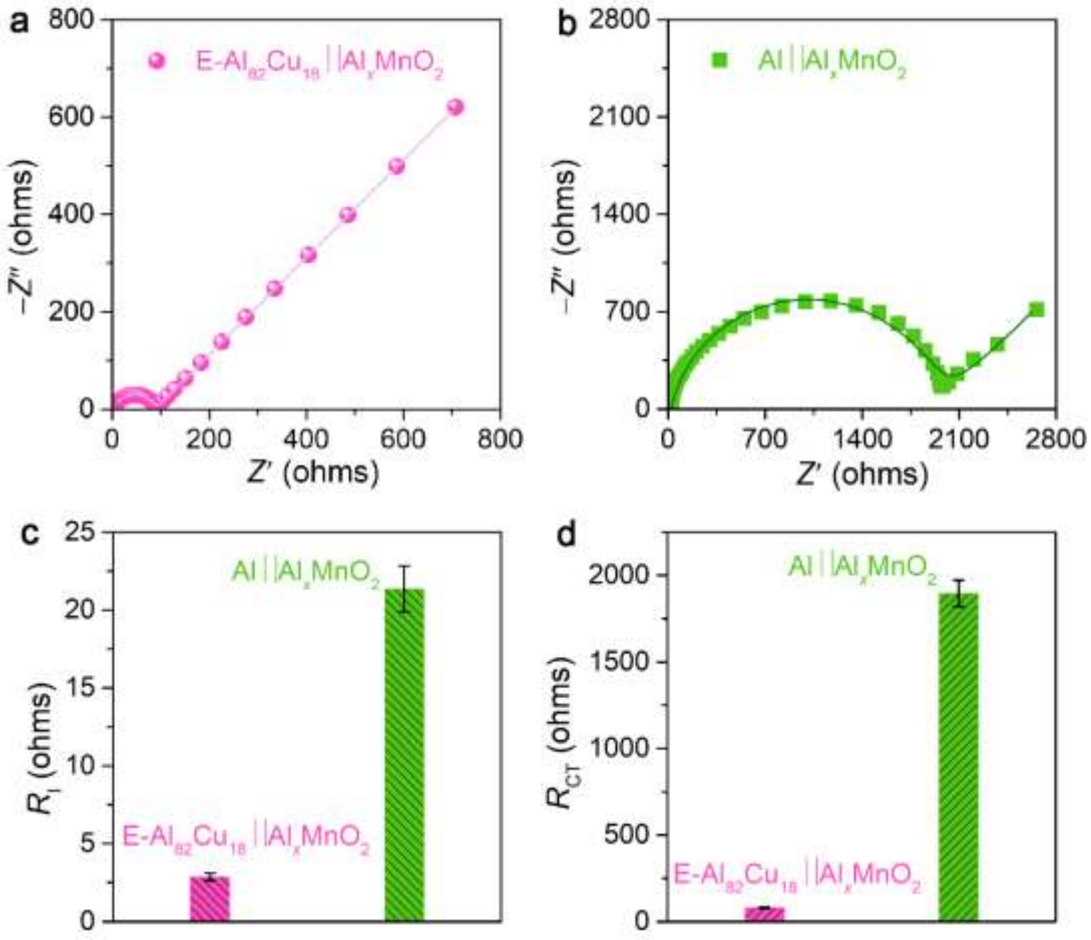

Supplementary Figure 22. Analysis of EIS spectra for full E-$Al_{82}Cu_{18}$||Al_xMnO_2 and Al||Al_xMnO_2 cells. a, EIS spectrum for E-$Al_{82}Cu_{18}$||Al_xMnO_2 cell. The pink sphere symbols are the raw data and the light pink line represents the fit data. **b,** EIS spectrum for Al||Al_xMnO_2 cell. The green square symbols are the raw data and the dark green line represents the fit data. **c**, **d**, Comparisons of the R_I (c) and R_{CT} (d) values of E-$Al_{82}Cu_{18}$||Al_xMnO_2 and Al||Al_xMnO_2 cells. Therein, both EIS spectra of E-$Al_{82}Cu_{18}$||Al_xMnO_2 and Al||Al_xMnO_2 cells are analyzed according to the equivalent circuit shown in Supplementary Figure 5d.

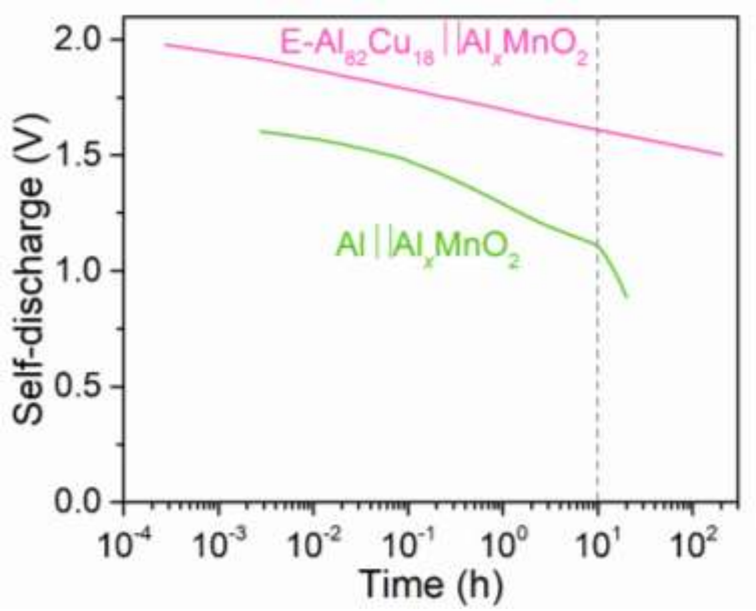

Supplementary Figure 23. Self-discharge behaviors of E-$Al_{82}Cu_{18}$||Al_xMnO_2 and Al||Al_xMnO_2 full cells.

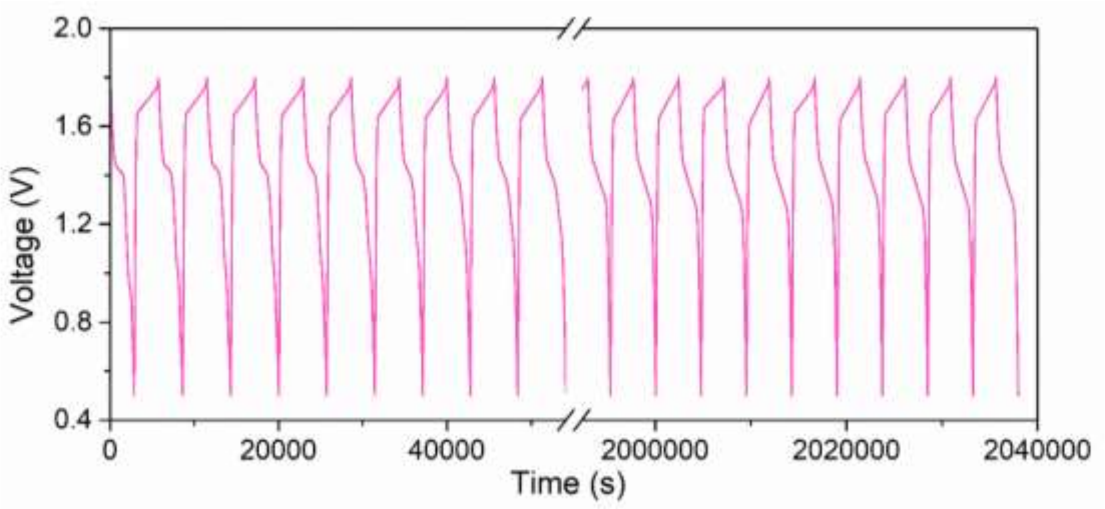

Supplementary Figure 24. Voltage profiles during the charge/discharge of E-$Al_{82}Cu_{18}$||Al_xMnO_2 full cell at specific current of 500 mA g^{-1}.

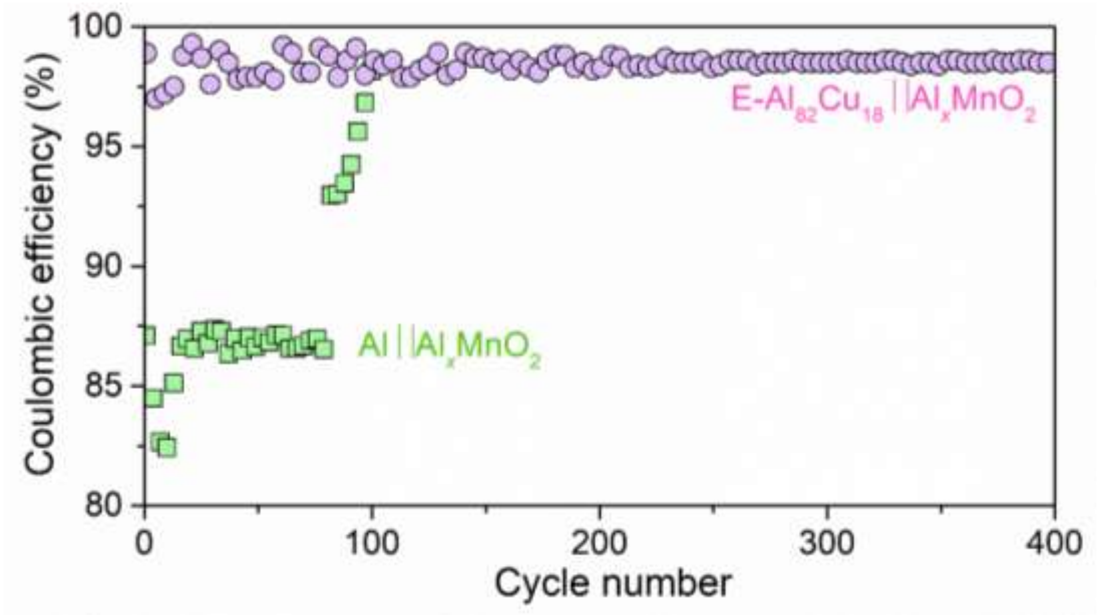

Supplementary Figure 25. Coulombic efficiency of E-$Al_{82}Cu_{18}$||Al_xMnO_2 and Al||Al_xMnO_2 full cells during the charge/discharge at specific current of 500 mA g^{-1}.

Supplementary Table 1. Abundance, volumetric capacity, gravimetric capacity, charge density of cations and cost for Al, Zn, K, Na and Li. Here the superscript numbers are reference numbers.

	Al	Zn	K	Na	Li
**Abundance** (%)	8.2 [1]	0.0075 [1]	2.1 [1]	2.3 [1]	0.0018 [1]
**Volumetric capacity** (mAh cm^{-3})	8056 [2,3]	5851 [2,3]	591 [2,3]	1128 [2,3]	2062 [2,3]
**Gravimetric capacity** (mAh g^{-1})	2981 [2,3]	820 [2,3]	685 [2,3]	1166 [2,3]	3861 [2,3]
**Charge density of cations** (C mm^{-3})	364 [4]	112 [4]	11 [4]	24 [4]	52 [4]
**Cost** (US$ T^{-1})	2297 [2,4]	2789 [2,4]	18370 [2,4]	3061 [2,4]	89557 [2,4]

Supplementary Table 2. O_2 concentrations in 2 M $Al(OTF)_3$ aqueous electrolytes that are treated by different methods.

**Methods and conditions for electrolyte treatments**	**O_2 concentrations (C_{O2}, mg L^{-1})**
Purging N_2 for 2 h	0.13 ± 0.005
Purging N_2 for 0.5 h	4.4 ± 0.05
As-prepared one	8.9 ± 0.05
Purging O_2 for 1 h	10.9 ± 0.05
Purging O_2 for 2 h	13.6 ± 0.05

Supplementary Table 3. The R_I and R_{CT} values in equivalent circuit models for EIS spectra of E-$Al_{82}Cu_{18}$, Al_2Cu and Al symmetric cells in 2 M $Al(OTF)_3$ with different O_2 concentrations.

C_{O2} (mg L^{-1})	E-$Al_{82}Cu_{18}$		Al_2Cu		Al	
	R_I (Ω)	R_{CT} (Ω)	R_I (Ω)	R_{CT} (Ω)	R_I (Ω)	R_{CT} (Ω)
**0.13**	3.24±0.24	161±8	7.40±0.63	292±12	30.7±2.0	3880±135
**4.4**	6.52±0.59	299±14	8.85±0.75	419±18	47.3±3.9	7367±289
**8.9**	9.70±0.67	442±16	9.68±0.73	522±24	43.5±2.4	10530±347
**10.9**	10.5±0.8	461±18	10.5±0.7	568±28	70.2±5.9	11530±466
**13.6**	11.9±1.1	517±19	11.7±0.7	687±21	85.8±6.3	13330±478

Supplementary Table 4. The R_I and R_{CT} values in equivalent circuit models for EIS spectra of E-$Al_{82}Cu_{18}$, Al_2Cu and Al symmetric cells in 2 M $Al(OTF)_3$ with C_{O2} =0.13 mg L^{-1} before and after Al stripping/plating for 240 or 24 h, respectively.

	**E-$Al_{82}Cu_{18}$**		**Al_2Cu**		**Al**	
	R_I (Ω)	R_{CT} (Ω)	R_I (Ω)	R_{CT} (Ω)	R_I (Ω)	R_{CT} (Ω)
**0 h**	3.24±0.24	161±8	7.40±0.63	292±12	30.7±2.0	3881±135
**240 h**	5.11±0.36	181±9	15.1±1.39	582±21		
**24 h**					36.1±2.6	8855±410

Supplementary Table 5. The ICP analysis of the as-prepared $Al_xMnO_2·nH_2O$.

**$Al_{0.12}MnO_2$**	**Al**	**Mn**
Molar concentration (mmol/L)	0.000771±0.000001	0.00632±0.00001
Atomic ratio (%)	10.87±0.03	89.13±0.03

Supplementary Table 6. Comparison of E-$Al_{82}Cu_{18}$||Al_xMnO_2 full cells with representative LIBs.

Anode/Cathode	Electrolyte	Battery type	Specific energy (Wh kg^{-1})	Average discharge capacity (mAh g^{-1})	Ref.
**Li|| $LiNi_{0.6}Mn_{0.2}Co_{0.2}O_2$**	LiFSI-DME-TTE	Nonaqueous Li-ion battery	667	185	5
**Sn-DGT||NCM622**	$LiPF_6$	Nonaqueous Li-ion battery	590	850	6
**Li ||NMC811**	LiFSI-DMTMSA	Nonaqueous Li-ion battery	758	205	7
**$Li_4Ti_5O_{12}$||$LiMn_2O_4$**	$LiN(SO_2CF_3)_2$	Aqueous Li-ion battery	331	135	8
**E-$Al_{82}Cu_{18}$||Al_xMnO_2**	$Al(OTF)_3$-H_2O	Aqueous Al-ion battery	672	470	This work

Supplementary Table 7. The R_I and R_{CT} values in equivalent circuit models for EIS spectra of E-$Al_{82}Cu_{18}$||Al_xMnO_2 and Al||Al_xMnO_2 AR-AMB cells.

**E-$Al_{82}Cu_{18}$//Al_xMnO_2**		**Al//Al_xMnO_2**	
R_I (Ω)	R_{CT} (Ω)	R_I (Ω)	R_{CT} (Ω)
2.84±0.24	86.0±4.2	20.4±1.5	1922±78

Supplementary Table 8. Comparison of aqueous and non-aqueous secondary AIBs.

Anode/Cathode	Electrolyte (mL)	Battery type	Specific Current (A g^{-1})	Cycles (*n*)	Capacity retention (%)	Coulombic efficiency (%)	Cell-level capacity (mAh g^{-1})	Cell-level specific energy (Wh kg^{-1})	Average discharge capacity (mAh g^{-1})	Average discharge potential (V)	Ref.
**Al‖graphite**	$AlCl_3$-[EMIm]Cl 1.5	Non-aqueous pouch-type	4	7500	100	98	19	27	65	1.42	9
**Al‖carbon paper**	$AlCl_3$-[EMIm]Cl 10	Non-aqueous teflon tank	0.1	100	100	100	20	28	70	1.4	10
**Al‖natural graphite**	$AlCl_3$-[EMIm]Cl n/a	Non-aqueous pouch-type	20	250000	70	99	21	29	96	1.38	11
**Al‖natural graphite**	$AlCl_3$-[EMIm]Cl n/a	Non-aqueous Swagelok-type	0.1	100	100	90	21	30	124	1.43	12
**Al‖graphene**	$AlCl_3$-[EMIm]Cl 0.05	Non-aqueous coin type	5	25000	97	98	22	31	97	1.41	13
**Al‖natural graphite flakes**	$AlCl_3$-[EMIm]Cl 2	Non-aqueous pouch-type	0.66	6000	100	99.5	23	32	60	1.39	14
**Al‖graphene film**	$AlCl_3$-[EMIm]Cl 0.2	Non-aqueous coin-type	100	250000	100	98	23	32	120	1.39	15
**Al‖3D graphene**	$AlCl_3$-[EMIm]Cl n/a	Non-aqueous pouch-type	5	10000	100	98	23	33	123	1.43	16
**Al‖PQ-triangle with graphite flakes**	$AlCl_3$-[EMIm]Cl n/a	Non-aqueous Swagelok-type	2	2000	50	99	35	50	84	1.43	17
**Al‖V_2CT_x**	$AlCl_3$-[EMIm]Cl n/a	Non-aqueous pouch-type	0.2	100	56	96	39	47	110	1.21	18

**Al||VO_2**	$AlCl_3$-[EMIm]Cl n/a	Non-aqueous coin type	0.05	100	73	99	43	26	125	0.6	19
**Al||V_2O_5**	$AlCl_3$-[EMIm]Cl n/a	Non-aqueous coin type	0.125	20	89	99	58	35	270	0.6	20
**Al||MoS_2-carbon nanofibres**	$AlCl_3$-[EMIm]Cl n/a	Non-aqueous pouch-type	0.1	200	43	95	47	47	155	1	21
**Al||Co_3S_4**	$AlCl_3$-[EMIm]Cl 0.05	Non-aqueous coin type	0.05	150	100	100	36	29	90	0.81	22
**Al||Co_9S_8 @CNT-CNF**	$AlCl_3$-[EMIm]Cl 0.035	Non-aqueous pouch-type	1	6000	56	96	45	45	100	1	23
**Al||Ni_3S_2**	$AlCl_3$-[EMIm]Cl n/a	Non-aqueous pouch-type	0.01	100	17	100	28	22	50	0.79	24
**Al||graphite**	$LiPF_6$, AlF_3EMC -DMC, VC n/a	Non-aqueous pouch-type	0.1	600	98	99	26	92	100	3.54	25
**Al||graphite**	$AlCl_3$-$6H_2O$ n/a	Aqueous coin type	0.5	1000	100	95	55	79	165	1.44	26
**Al||$Al_xMnO_2 \cdot nH_2O$**	$Al(OTF)_3$-H_2O n/a	Aqueous coin type	0.03	20	58	85	42	46	409	1.1	27
**IL-treated Al||α-MnO_2**	$Al(OTF)_3$-H_2O n/a	Aqueous coin type	0.1	40	44	100	41	54	250	1.32	28
**IL-treated Al||Birnessite-MnO_2**	$Al(OTF)_3$-H_2O $MnSO_4$ n/a	Aqueous coin type	0.1	25	58	99	33	37	470	1.12	29
**E-$Al_{82}Cu_{18}$||Al_xMnO_2**	$Al(OTF)_3$-H_2O 0.25	Aqueous coin type	0.2	200	82	99	66.7	90.2	470	1.35	This work

Supplementary References

1. https://environmentalchemistry.com/yogi/periodic/.
2. Zhang, Y., Liu, S., Ji, Y., Ma, J. & Yu, H. Emerging nonaqueous aluminum-ion batteries: challenges, status, and perspectives. *Adv. Mater.* **30**, 1706310 (2018).
3. Liang, Y., Dong, H., Aurbach, D. & Yao, Y. Current status and future directions of multivalent metal-ion batteries. *Nat Energy* **5**, 646-656 (2020).
4. Faegh, E., Ng, B., Hayman, D. & Mustain, W. Practical assessment of the performance of aluminum battery technologies. *Nat. Energy* **6**, 21-29 (2021).
5. Niu, C., Liu, D., Lochala, J.A., Anderson, C.S., Cao, X., Gross, M.E., Xu, w., Zhang, J.G., Whittingham, M.S., Xiao, J. & Liu, J. Balancing interfacial reactions to achieve long cycle life in high-energy lithium metal batteries. *Nat Energy* **6**, 723–732 (2021).
6. Mo, R., Tan, X., Li, F., Tao, R., Xu, J., Kong, D., Wang, Z., Xu, B., Wang, X., Wang, C., Li, j., Peng, Y. & Lu, Y. Tin-graphene tubes as anodes for lithium-ion batteries with high volumetric and gravimetric energy densities. *Nat Commun* **11**, 1374 (2020).
7. Xue, W., Huang, M., Li, Y. Zhu, Y., Gao, R., Xiao, X., Zhang, W., Li, S., Xu, G., Yu, Y., Li, P., Lopez, J., Yu, D., Dong, Y., Fan, W., Shi, Z., Xiong, R., Sun, C., Hwang, I., Lee, W., Horn, Y., Johnson, J.A. & Li, J. Ultra-high-voltage Ni-rich layered cathodes in practical Li metal batteries enabled by a sulfonamide-based electrolyte. *Nat Energy* **6**, 495–505 (2021).
8. Shang, Y., Chen, N., Li, Y., Chen, S., Lai, J., Huang, Y., Qu, W., Wu, F. & Chen, R. An "Ether-In-Water" Electrolyte Boosts Stable Interfacial Chemistry for Aqueous Lithium-Ion Batteries. *Adv. Mater.* **32**, 2004017 (2020).
9. Lin, M.C., Gong, M., Lu, B., Wu, Y., Wang, D.Y., Guan, M., Angell, M., Chen, C., Yang, J., Hwang, B.J. & Dai, H. An ultrafast rechargeable aluminium-ion battery. *Nature* **520**, 324-328 (2015).
10. Sun, H., Wang, W., Yu, Z., Yuan, Y., Wang, S. & Jiao, S. A new aluminium-ion battery with high voltage, high safety and low cost. *Chem. Commun.* **51**, 11892-11895 (2015).
11. Elia, G.A., Kyeremateng, N.A., Marquardt, K. & Hahn, R. An aluminum/graphite battery with ultra-high rate capability. *Batter. Supercaps.* **2**, 83-90 (2019).
12. Kravchyk, K.V., Wang, S., Piveteau, L. & Kovalenko, M.V. Efficient aluminum chloride–natural graphite battery. *Chem. Mater*. **29**, 4484-4492 (2017).
13. Chen, H., Guo, F., Liu, Y., Huang, T., Zheng, B., Ananth, N., Xu, Z., Gao, W. & Gao, C. A defect-free principle for advanced graphene cathode of aluminum-ion battery. *Adv. Mater.* **29**, 1605958 (2017).
14. Wang, D.Y., Wei, C.H., Lin, M.C., Pan, C.J., Chou, H.L., Chen, H.A., Gong, M.,

Wu, Y., Yuan, C., Angell, M., Hsieh, Y.-J., Chen, Y.H., Wen, C.Y., Chen, C.W., Hwang, B.J., Chen, C.C. & Dai, H. Advanced rechargeable aluminum ion battery with a high-quality natural graphite cathode. *Nat. Commun*. **8**, 14283 (2017).

15. Chen, H., Xu, H., Wang, S., Huang, T., Xi, J., Cai, S., Guo, F., Xu, Z., Gao, W. & Gao, C. Ultrafast all-climate aluminum-graphene battery with quarter-million cycle life. *Sci. Adv.* **3**, eaao7233 (2017).
16. Yu, X., Wang, B., Gong, D., Xu, Z. & Lu, B. Graphene nanoribbons on highly porous 3D graphene for high-capacity and ultrastable Al-ion batteries. *Adv. Mater.* **29**, 1604118 (2017).
17. Kim, D. J., Yoo, D.J., Otley, M.T., Prokofjevs, A., Pezzato, C., Owczarek, M., Lee, S.J., Choi, J.W. & Stoddart, F. Rechargeable aluminium organic batteries. *Nat. Energy* **4**, 51 (2019).
18. VahidMohammadi, A., Hadjikhani, A., Shahbazmohamadi, S. & Beidaghi, M. Two-dimensional vanadium carbide (MXene) as a high-capacity cathode material for rechargeable aluminum batteries. *ACS Nano* **11**, 11135-11144 (2017).
19. Wang, W., Jiang, B., Xiong, W., Sun, H., Lin, Z., Hu, L., Tu, J., Hou, J., Zhu, H. & Jiao, S. A new cathode material for super-valent battery based on aluminium ion intercalation and deintercalation. *Sci. Rep*. **3**, 3383 (2013).
20. Jayaprakash, N., Das, S. & Archer, L. The rechargeable aluminum-ion battery. *Chem. Commun.* **47**, 12610–12612 (2011).
21. Yang, W., Lu, H., Cao, Y., Xu, B., Deng, Y. & Cai, W. Flexible free-standing MoS_2/carbon nanofibers composite cathode for rechargeable aluminum-ion batteries. *ACS Sustain. Chem. Eng.* **7**, 4861-4867 (2019).
22. Li, H., Yang, H., Sun, Z., Shi, Y., Cheng, H. & Li, F. A highly reversible Co_3S_4 microsphere cathode material for aluminum-ion batteries. *Nano Energy* **56**, 100-108 (2019).
23. Hu, Y., Ye, D., Luo, B., Hu, H., Zhu, X., Wang, S., Li, L., Peng, S. & Wang, L. A binder-free and free-standing cobalt sulfide@carbon nanotube cathode material for aluminum-ion batteries. *Adv. Mater*. **30**, 1703824 (2018).
24. Wang, S., Yu, Z., Tu, J., Wang, J., Tian, D., Liu, Y. & Jiao, S., A novel aluminum-ion battery: Al/AlCl3-[EMIM]Cl/Ni3S2@graphene. *Adv. Energy Mater*. **6,** 1600137 (2016).
25. Wang, S., Jiao, S., Tian, D., Chen, H., Jiao, H., Tu, J., Liu, Y. & Fang, D. A novel ultrafast rechargeable multi-ions battery. *Adv. Mater*. **29**, 1606349 (2017).
26. Pan, W., Wang, Y., Zhang, Y., Kwok, H., Wu, M., Zhao, X. & Leung, D.Y.C. A low-cost and dendrite-free rechargeable aluminium-ion battery with superior performance. *J. Mater. Chem. A* **7**, 17420–17425 (2019).
27. Wu, C., Gu, S., Zhang, Q., Bai, Y., Li, M., Yuan, Y., Wang, H., Liu, X., Yuan, Y., Zhu, N., Wu, F., Li, H., Gu, L. & Lu, J. Electrochemically activated spinel

manganese oxide for rechargeable aqueous aluminum battery. *Nat. Commun.* **10**, 73 (2019).

28. Zhao, Q., Zachman, M.J., Al Sadat, W., Zheng, J., Kourkoutis, L.F. & Archer, L. Solid electrolyte interphases for high-energy aqueous aluminum electrochemical cells. *Sci. Adv.* **4**, eaau8131 (2018).
29. He, S., Wang, J., Zhang, X., Chen, J., Wang, Z., Yang, T., Liu, Z., Liang, Y., Wang, B., Liu, S., Zhang, L., Huang, J., Huang, J., O'Dell, L.A. & Yu, H. A high-energy aqueous aluminum-manganese battery. *Adv. Funct. Mater.* **29**, 1905228 (2019).
